# Supplementary material for: A disease-linked lncRNA mutation in RNase MRP inhibits ribosome synthesis
Source: Nat Commun. 2022 Feb 3;13:649. doi: 10.1038/s41467-022-28295-8 (PMC8814244; doi:10.1038/s41467-022-28295-8)
Supplement: Supplementary file 1 — Supplementary Information [file 41467_2022_28295_MOESM1_ESM.pdf]

Supplementary Figures

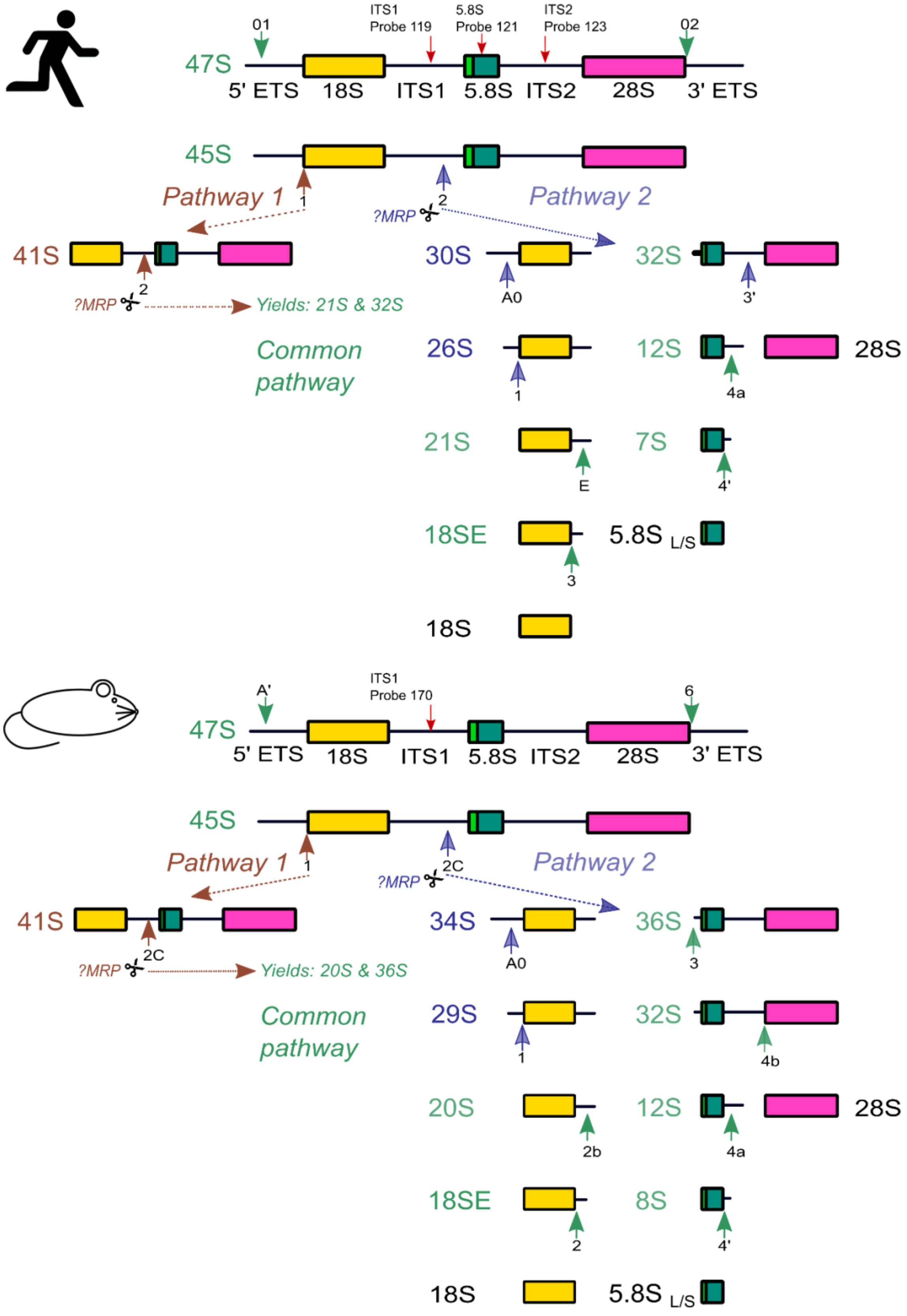

Supplementary Figure 1: Pre-rRNA processing in humans and mice. Ribosomes are composed of ribosomal RNA (rRNA) and ribosomal proteins, and consist of a large subunit (LSU) and small subunit (SSU). 18S is the rRNA for the small ribosomal subunit. 5.8 and 28S are rRNAs in the large subunit. RNA encoding the mature 5.8S, 18S and 28S rRNA are transcribed together as a long precursor, called 47S in humans and mice. Both ends of 47S are flanked by external transcribed spacers (5' ETS and 3' ETS), and two internal transcribed spacers (ITS) separate the mature species. To make functional ribosomes, these spacers are removed in a complex series of processing steps. In humans, 47S is first processed by trimming the 5' and 3' ETS, by cleavage at sites 01 and 02, yielding the 45S precursor. After this, two distinct processing pathways have been described. Pathway 1 generates the 41S species by removing the remaining 5' ETS, while the LSU and SSU precursors remain joined. In Pathway 2, the initial cleavage occurs at site 2 which separates the LSU and SSU. The most likely candidate for mediating this site 2 cleavage is RNase MRP[1]. The 41S species in Pathway 1 is also cleaved at site 2, yielding the 21S and 32S precursors which are then processed similarly to the same species generated in Pathway 2. In mice, pre-rRNA processing follows a similar pattern. The equivalent of site 2 is termed site 2C. In all studied fungi, animal and plants, 5.8S rRNA is present in both long and short forms, where the long form has a 7 to 8 nucleotide 5' extension. The biological significance of the two forms is not known. The processing pathways depicted here are simplified for clarity, but show the major pre-rRNA species detected in experiments in this study. Solid green arrows indicate cleavage/processing at named sites in the common pathway. Brown and blue solid arrows represent cleavage/processing steps exclusive to Pathway 1 and Pathway 2, respectively. Dotted arrows indicate direction of processing. Red arrows indicate position of northern probes used in this study.

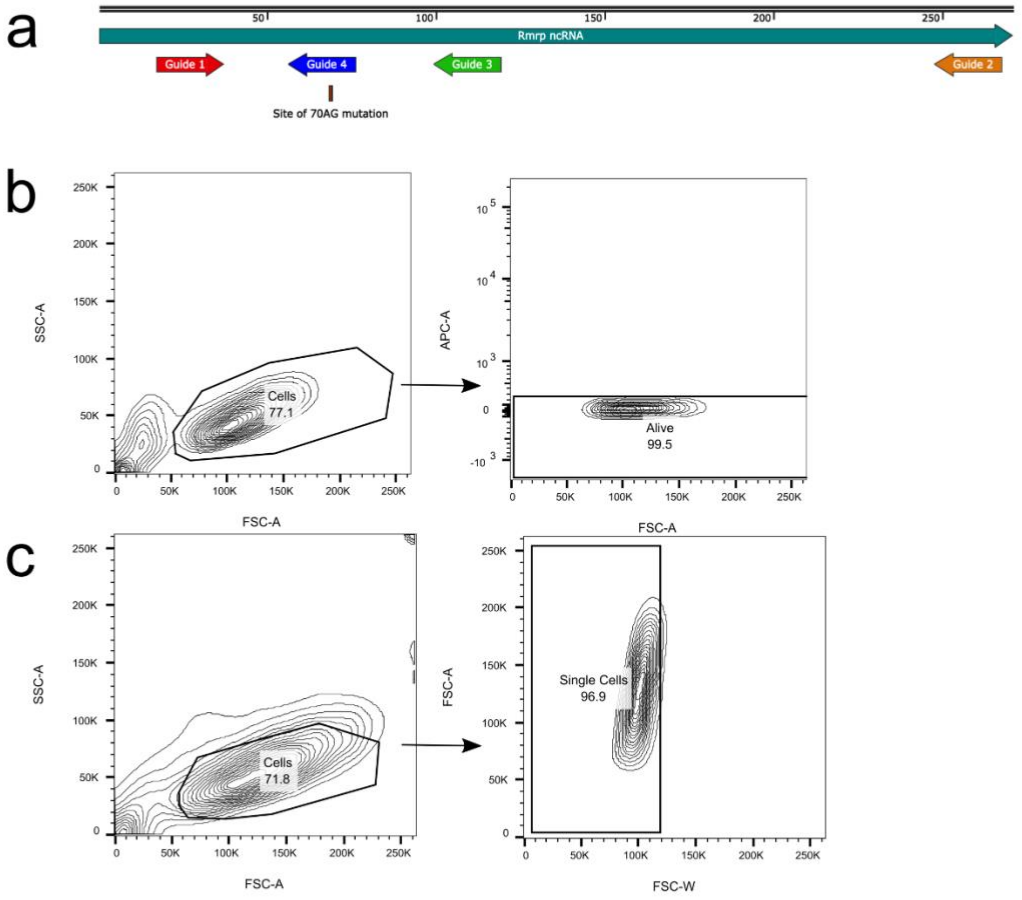

Supplementary Figure 2: Extended data related to Figures 1 and 3: (A) Position of CRISPR guides targeting murine *Rmrp* used in primary mouse T cell CRISPR experiments. (B) Flow cytometry gating for mouse T cell experiments: cells were gated first on morphological lymphocytes, then live cells as assessed by Zombie Red live-dead stain on the APC channel. (C) Gating for FlowFish experiments: K562 cells were gated first on morphologically live cells then on single cells.

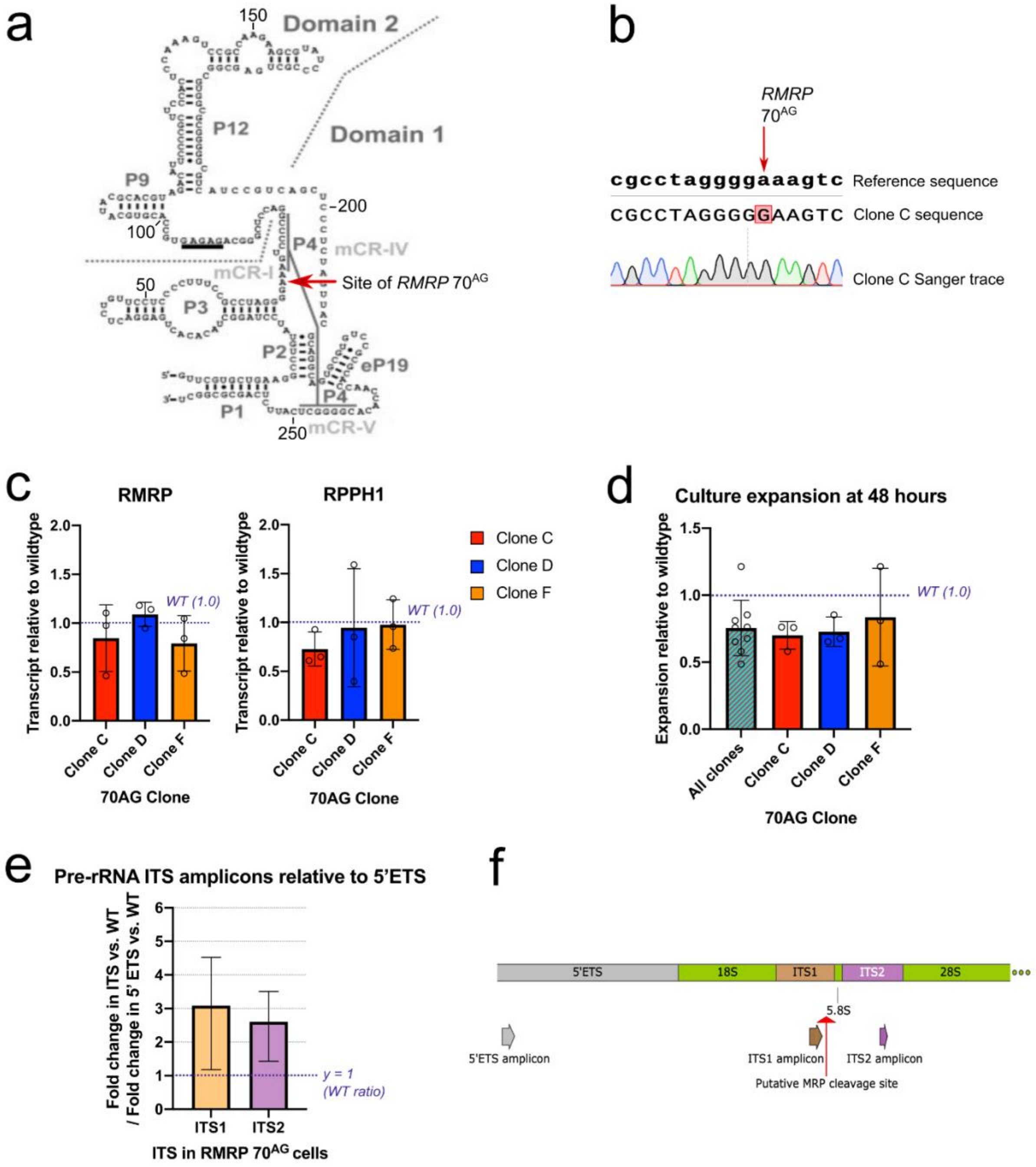

Supplementary Figure 3: CRISPR-Cpf1 allows generation of K562 lines homozygous for *RMRP* 70^{AG} mutation. A: Predicted secondary structure of human *RMRP* showing site of 70^{AG} mutation. Adapted from Esakova and Krasilnikov[2]**.** Regions evolutionarily conserved from M-type archaeal RNase P RNA are indicated as mCR-I, mCR-IV and mCR-V. Named base-paired RNA stems are indicted as P1, P2, etc. B: Extract from Sanger sequencing trace for *RMRP* 70^{AG} clone C. Similar results were obtained for the other clones used in this study. C: qPCR to quantify the RNase MRP ncRNA (RMRP) and the RNase P ncRNA (RPPH1*)* in *RMRP* 70^{AG} clones. Mean and SD of results from three independent

experiments, each normalized first to a house-keeping gene product (HPRT*)* and then the average of wildtype values obtained in that experiment. D: Expansion of *RMRP* 70^{AG} and wildtype K562 cells after 48 hours of culture. Cells were plated at a density of 1 x 10^5 / mL, in triplicate, and either counted manually every 24 hours, or density tracked with live cell imaging software (Incucyte, version IncuCyte2011A). Graph contains results obtained from one manual experiment and two live-cell imaging replicates (mean and SD). E: Relative abundance of ITS vs 5' ETS in *RMRP* 70^{AG} cells. The pre-rRNA regions indicated in (F) were amplified by qPCR. The CT value for each amplicon was first normalized to a house-keeping gene (B2M)*,* then fold change for mutant cells vs. wildtype calculated. Finally, the ratio of fold change for ITS vs 5'ETS amplicons was calculated. Contains data from three independent experiments, each including samples from three different CRISPR clones (total of 14 mutant samples for ITS1 and 15 for ITS2; median and 95% confidence interval). F: qPCR amplicons across 47S pre-rRNA, as used in (E). Putative cleavage site is taken from analyses reported in[1]. Source data are provided as a Source Data file.

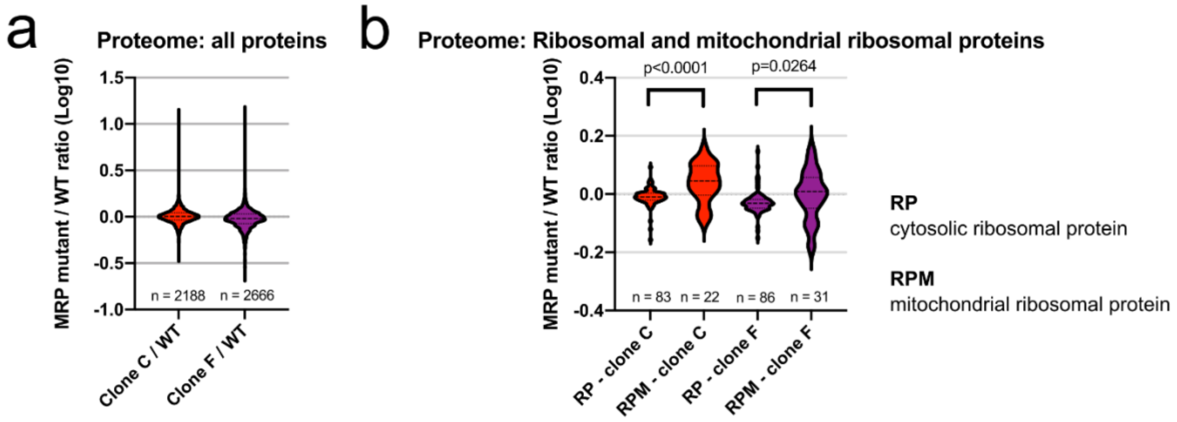

Supplementary Figure 4: Proteome of *RMRP* 70^{AG} cells. A: Log-transformed SILAC ratios (70^{AG} mutant / wildtype cells) for all proteins quantified in two SILAC mixes using independent CRISPR clones. Lysates from indicated cells were grown in heavy or light SILAC media, mixed 1:1 by protein abundance and electrophoresed on a polyacrylamide gel. Proteins were digested in-gel to release peptides for mass spectrometry. B: Log-transformed SILAC ratios for cytosolic ribosome proteins (RP) and mitochondrial ribosome proteins (RPM), obtained in two SILAC mixes. Violin plots depict distribution of ratios, with lines at median and quartiles. Indicated p-values derived from two-tailed t tests: for clone C, t=4.896, df=103; for clone F, t=2.249, df=115. Source data are provided as a Source Data file.

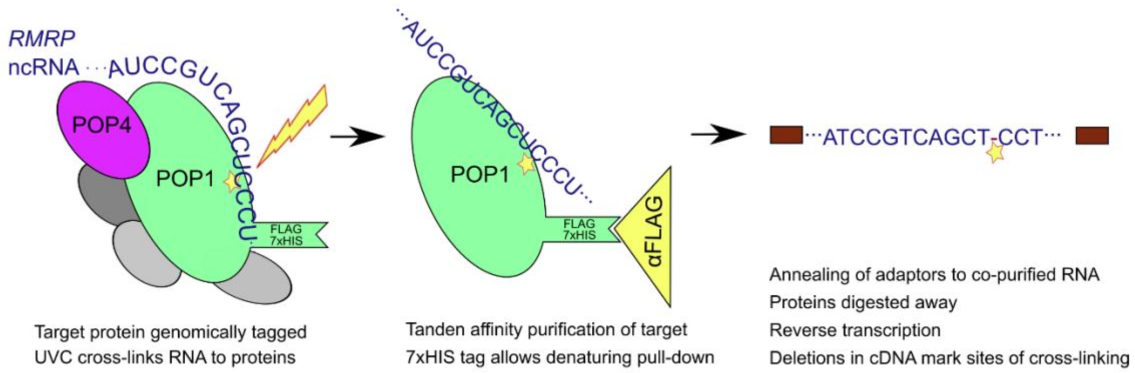

Supplementary Figure 5: Schematic overview of CRAC technique. Genomic copies of genes encoding proteins of interest were modified to encode a bipartite FLAG-7xHIS tag. Cells were UV irradiated to induce RNA:protein cross-links. The target protein was then purified under stringent, denaturing conditions. Adaptors were annealed to co-purified RNA, which was then reverse transcribed and a library prepared for sequencing. Sites of RNA:protein cross-links are indicated in the data by single nucleotide deletions.

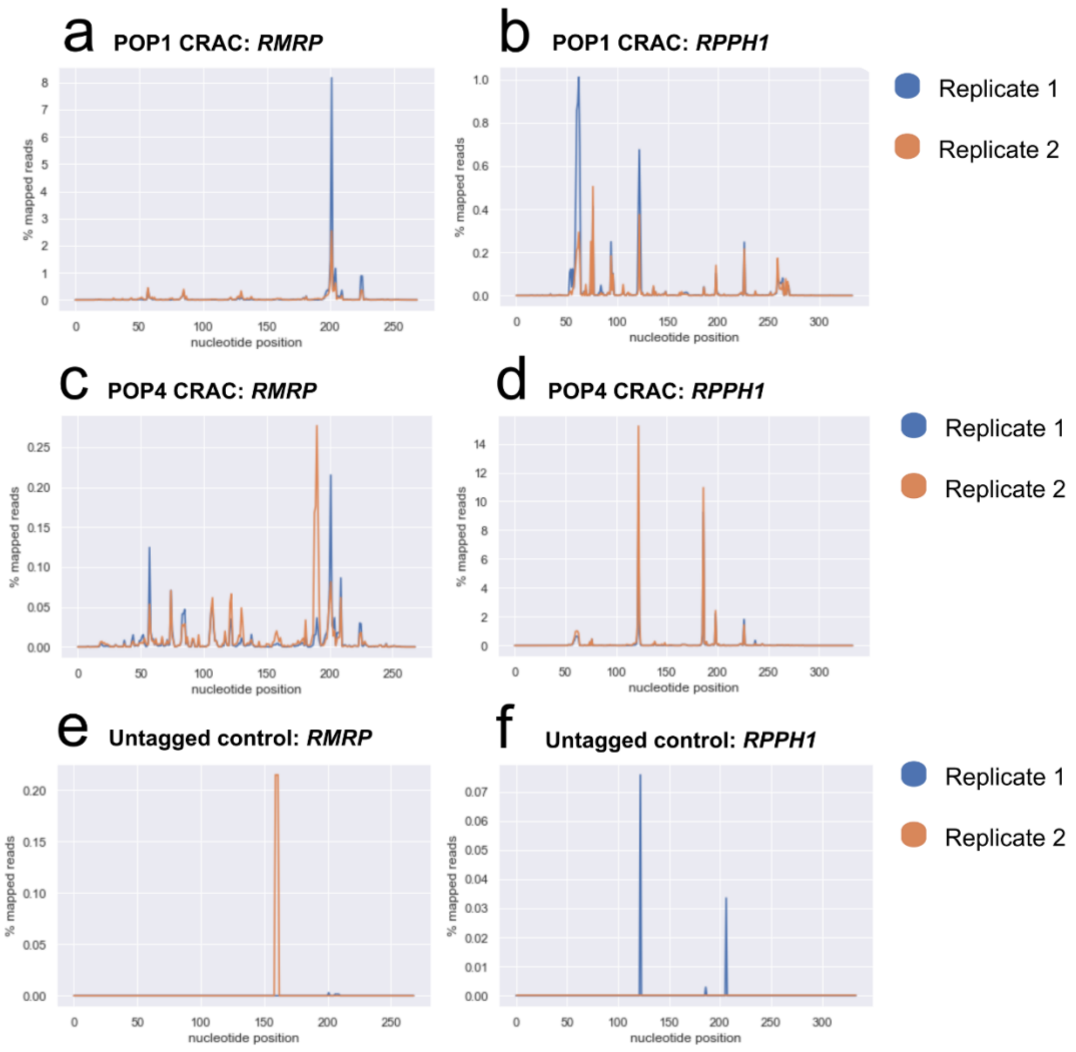

Supplementary Figure 6: RNA:protein interactions in human MRP/P complexes. Sites of deletions, indicating RNA:protein crosslink sites, between human MRP/P complex proteins and ncRNAs. RMRP is the RNase MRP ncRNA. RPPH1 is the RNase P ncRNA. A and B: Deletions in RMRP and RPPH1 in POP1 CRAC. C and D: Deletions in RMRP and RPPH1 in POP4 CRAC. E and F: Deletions in RMRP and RPPH1 in negative control (untagged cells) CRAC. Graphs show proportion of mapped reads in two independent experiments. Note differences in scales used. Source data are provided as processed data files.

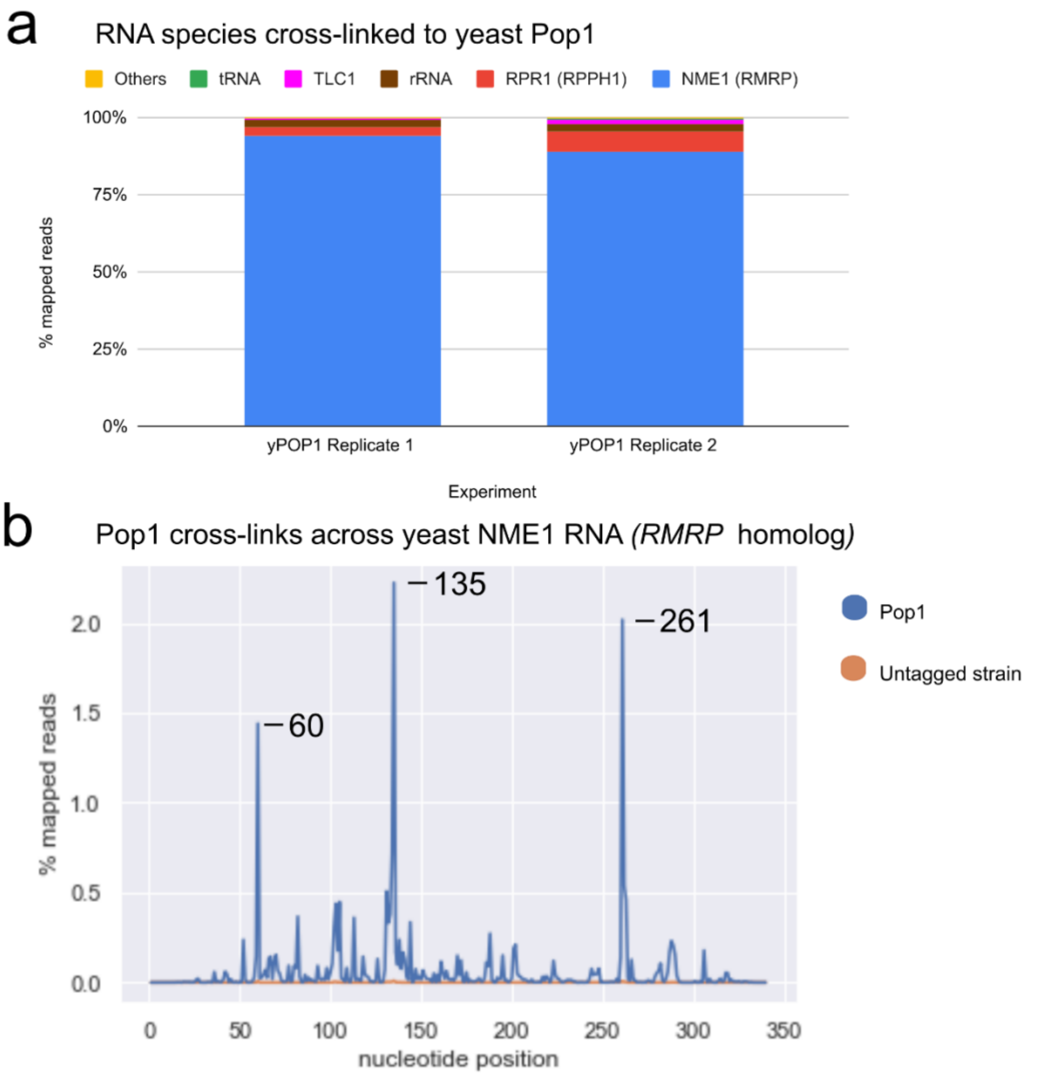

Supplementary Figure 7: RNA species interacting with yeast Pop1 protein. A: RNA species and biotypes recovered in yeast Pop1 CRAC experiments. Graphs show relative proportions of mapped reads in two independent experiments. TLC1 is the ncRNA component of the telomerase complex, a known Pop1 interactor in yeast[3]. RPR1 is the yeast RNase P RNA, and NME1 is the RNase MRP RNA. B: Reproducible cross-link sites between Pop1 protein and the RNase MRP RNA NME1. Labelled numbers represent the nucleotide position of each peak. Source data are provided as a Source Data file (panel A) and as processed data files (panel B).

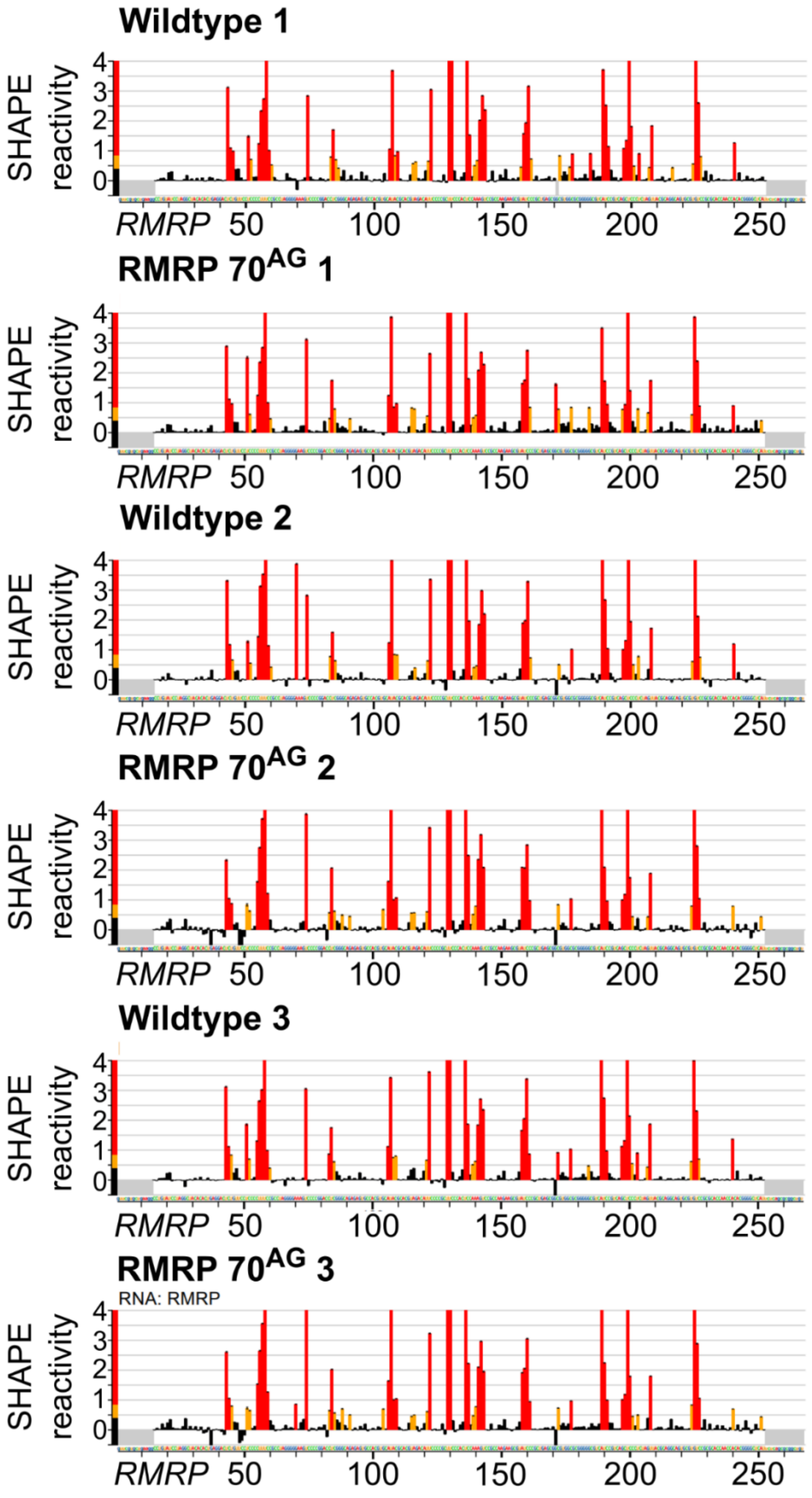
Wildtype 1
RMRP 70AG 1
Wildtype 2
RMRP 70AG 2
Wildtype 3
RMRP 70AG 3
RNA: RMRP
SHAPE reactivity
RMRP
50
100
150
200
250

Supplementary Figure 8: In-cell SHAPE-MaP profiles for wildtype and *RMRP* 70^{AG} cells.

SHAPE-MaP profiles for RMRP in wildtype and *RMRP* 70^{AG} cells. *In vivo* selective 2′-hydroxyl acylation of RNA was induced by adding 1M7 to the culture medium. RNA was extracted and reverse transcribed. Sites showing higher nucleotide-misincorporation frequency (indicating chemical modification) were determined by cDNA sequencing. Higher SHAPE reactivity scores indicate greater flexibility at that nucleotide Three biologically independent pairs are shown. Differences in reactivity profiles calculated by the deltaSHAPE tool[4] are shown in Supplementary Fig. 9. Positions showing consistent changes between replicates are annotated in Fig. 5E. Source data are provided as processed data files.

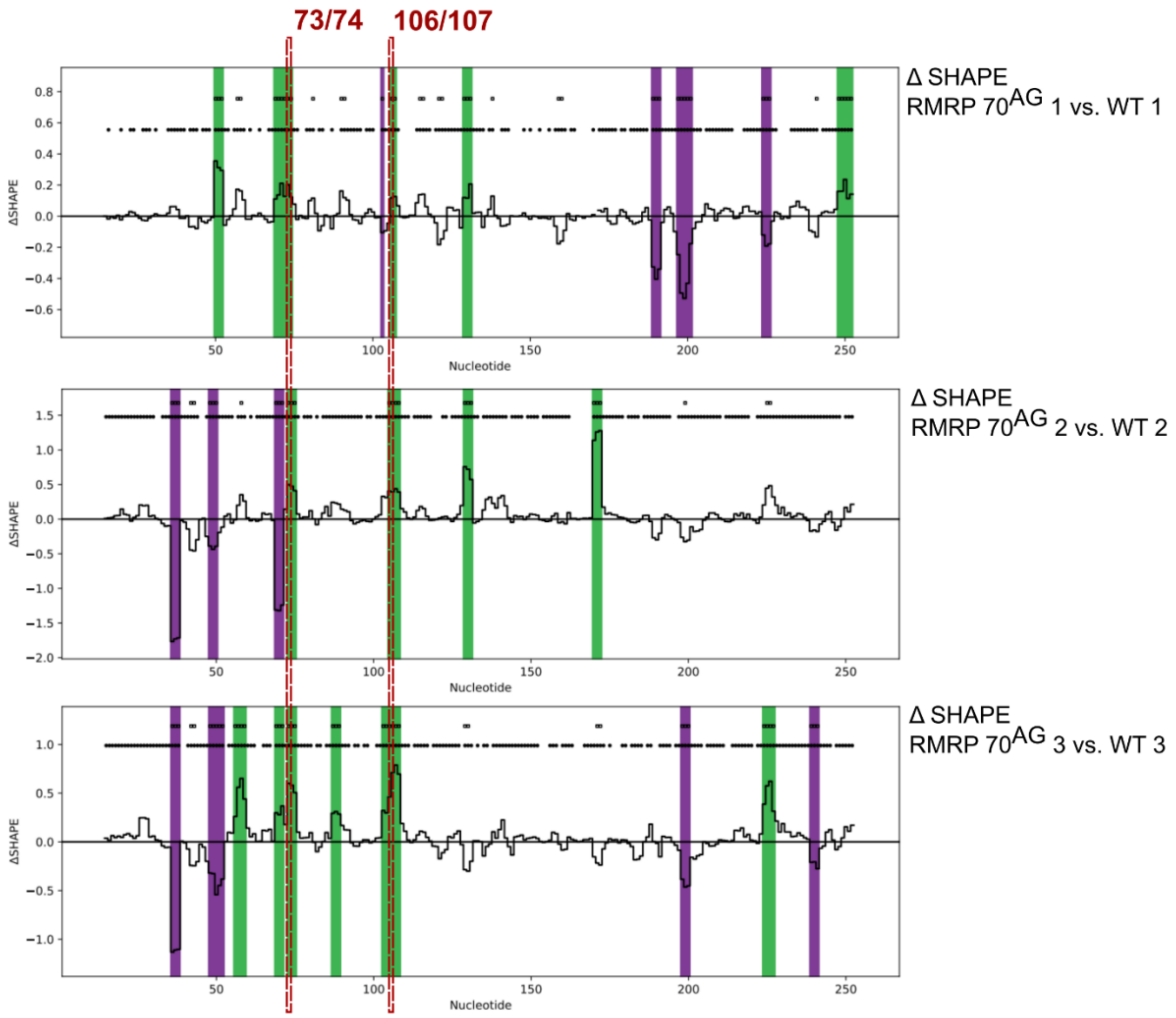

Supplementary Figure 9: deltaSHAPE profiles comparing wildtype and *RMRP* 70^{AG} SHAPE-MaP profiles. Statistical comparison of the SHAPE-MaP profiles as shown in Supplementary Fig. 8 were performed with the deltaSHAPE tool[4]. Regions with statistically significant increases or decreases in reactivity are highlighted and green and purple, respectively. Regions where these changes were consistent across replicates are highlighted with a dashed red box marked with nucleotide numbers and annotated in Fig. 5E. Source data are provided as processed data files.

Supplementary Tables

Supplementary Table 1: Guide RNAs used to target Rmrp in mouse T cells

Name	Sequence
NR234-mRMRP-KO-G1	GTTTCCTAGGCTACATACGA
NR235-mRMRP-KO-G2	GCCAAGAAGCGACCCCTCCG
NR236-mRMRP-KO-G3	TGGCTCGCACCAACCACACG
NR237-mRMRP-KO-G4	GGGGAAAGTCCCCGGACCAC

Supplementary Table 2: Oligonucleotide probes used for Northern Blotting experiments

Probe	Species	Target	Sequence	Reference
119	Human	ITS1	AGGGGTCTTTAAACCTCCGCGCCGGAA CGCGCTAGGTAC	[5]
121	Human	5.8S rRNA	CAATGTGTCCTGCAATTCAC	[5]
123	Human	ITS2	GCGCGACGGCGGACGACACCGCGGCG TC	[5]
170	Mouse	ITS1	TTCTCTCACCTCACTCCAGACACCTCG CTCCACA	[6]

Supplementary Table 3: Components used for CRISPR-mediated editing of human cells

Target	Item	Sequence
POP1 (N terminal tag)	ssODN	TCCCTGTATTAAATGTATTACTTCCTTTCCAGAAATGGAT TACAAAGACGACGATGACAAGGATTATAAGGACGACGA TGATAAGGACTACAAGGACGACGACGATAAGGCACTTT TGGAGGTTCTCTTCCAAGGCCCTGCCTCCGGCCATCAC CACCACCACCATATGTCAAATGCAAAAGAAAGAAAACAC GCCAAGA
POP1 (N terminal tag)	Guide	TGCATTTGACATTTCTGGAAAGG
POP1 (N terminal tag)	Fw primer	TCATTGATTCAGGTGGCTCTT
POP1 (N terminal tag)	Rv primer	AGTCACATTGGTAGGCTGGT
POP4 (C terminal tag)	ssODN	ATTCCAGCTTCGGTCAAGTGAACGGTCTGCGAAGAAGT TCAAAGCGAAGGGAACGATTGACCTGTCGGGCGGACAT CACCACCATCATCACCATCACGCCGCAGCCGCAGATTA TAAAGATGACGATGACAAGTGAATTCTTTGCCGTCTAAG GCAGTTGTTTATGACAGCTGAAAACTGGACACTCCCTAA ATGTCCAC
POP4 (C terminal tag)	Guide	TTAGACGGCAAAGAATTCAC
POP4 (C terminal tag)	Fw primer	TTCCAGCTTCGGTCAAGTGAA
POP4 (C terminal tag)	Rv primer	TCAACTCGGAGCGTCACTG
RMRP (70^{AG})	ssODN	TTGGAGTGGGAAGCGGGGAATGTCTACGTGCGTATGCA CGTGGCACTCTCTGCCCGAGGTCCGGGGACTTCCCCCT AGGCGGAAAGGGGAGGAACAGAGTCCTCAGTGTGTAG CCTAGGATACAGGCCTTCAGCACGAAC
RMRP (70^{AG})	Guide	CCCTAGGCGGAAAGGGGAGG
RMRP (70^{AG})	Fw primer	AATCTCACGCCACCAACTTT
RMRP (70^{AG})	Rv primer	GGAGGTCGAGGCTGCAGT

Supplementary Table 4: Primers used for HIS-TEV-Protein A tagging of yeast Pop1

Target	Forward primer	Reverse primer
Pop1	GGTACCAACACTTATAGGTT GGGGGAGTGGTCGAAAATAT CCGTAGAGCACCATCACCAT CAC	CTTTATAGGATATCGGTCGTACATA TAATTCAGTTCAGTTCATTAACGAC TCACTATAGGGCGA

Supplementary Table 5: Primers used for qPCR experiments on human cell lines

Amplicon	Forward primer	Reverse primer
U1	GGGAGATACCATGATCACGAAGGT	CCACAAATTATGCAGTCGAGTTTCCC
RMRP	CGTAGACATTCCCCGCTTCC	GCGTAACTAGAGGGAGCTGAC
RPPH1	AGCTTGGAACAGACTCACGG	AATGGGCGGAGGAGAGTAGT
HPRT	GGACAGGACTGAACGTCTTGC	CTTGAGCACACAGAGGGCTACA
B2M	ATGGAGGTTTGAAGATGCC	CTAAGTTGCCAGCCCTCCT
5’ETS	GTGCGTGTCAGGCGTTCT	GGGAGAGGAGCAGACGAG
ITS1	GACCCCTTGGGGGGATCG	CGCGGACACCACCCCACA
ITS2	CCCGCCCCGCGGCCCGC	CGACGCGGAAGCTCGGGA

Supplementary Table 6: rRNA probes used for FlowFISH experiments

Probe	Sequence
18S	TTTACTTCCTCTAGATAGTCAAGTTCGACC
18S - scrambled	ACCTTCATTCTCGTAATCCGTTCGAAGTTA
28S	CCCGTTCCCTTGGCTGTGGTTTCGCTAGATA
28S - scrambled	ATTCGTTAGCTCGTCTCTTGCGGATCCTCGG

Supplementary Table 7: primers used for in-cell SHAPE-MaP experiments on human RMRP

Name	Sequence	Description
NRs1-MRP-RT	ACAGCCGCGCTGAGA	RT primer for human RMRP SHAPE
NRs2-MRP-PCR1-FW	GACTGGAGTTCAGACGTGTGCTCTTCCGATCTNNNNNGTTCGTGCTGAAGGC	Step 1 PCR primer for human RMRP SHAPE
NRs3-MRP-PCR1-RW	CCCTACACGACGCTCTTCCGATCTNNNNNACAGCCGCGCTGAGA	Step 1 PCR primer for human RMRP SHAPE
NRs4-PCR2-RW	AATGATACGGCGACCACCGAGATCTACACTCTTTCCCTACACGACGCTCTTCCG	Universal reverse primer
SHAPE-PCR2-FW1	CAAGCAGAAGACGGCATACGAGATCGTGATGTGACTGGAGTTCAGAC	Barcoded forward primer
SHAPE-PCR2-FW2	CAAGCAGAAGACGGCATACGAGATACATCGGTGACTGGAGTTCAGAC	Barcoded forward primer
SHAPE-PCR2-FW3	CAAGCAGAAGACGGCATACGAGATGCCTAAGTGACTGGAGTTCAGAC	Barcoded forward primer
SHAPE-PCR2-FW4	CAAGCAGAAGACGGCATACGAGATTGGTCAGTGACTGGAGTTCAGAC	Barcoded forward primer
SHAPE-PCR2-FW5	CAAGCAGAAGACGGCATACGAGATCACTGTGTGACTGGAGTTCAGAC	Barcoded forward primer
SHAPE-PCR2-FW6	CAAGCAGAAGACGGCATACGAGATATTGGCGTGACTGGAGTTCAGAC	Barcoded forward primer
SHAPE-PCR2-FW7	CAAGCAGAAGACGGCATACGAGATGATCTGGTGACTGGAGTTCAGAC	Barcoded forward primer
SHAPE-PCR2-FW8	CAAGCAGAAGACGGCATACGAGATTCAAGTGTGACTGGAGTTCAGAC	Barcoded forward primer
SHAPE-PCR2-FW9	CAAGCAGAAGACGGCATACGAGATCTGATCGTGACTGGAGTTCAGAC	Barcoded forward primer
SHAPE-PCR2-FW10	CAAGCAGAAGACGGCATACGAGATAAGCTAGTGACTGGAGTTCAGAC	Barcoded forward primer
SHAPE-PCR2-FW11	CAAGCAGAAGACGGCATACGAGATGTAGCCGTGACTGGAGTTCAGAC	Barcoded forward primer
SHAPE-PCR2-FW12	CAAGCAGAAGACGGCATACGAGATTACAAGGTGACTGGAGTTCAGAC	Barcoded forward primer

Supplementary Table 8: Buffers used for human CRAC experiments

Buffer	Composition
**LB**	50 mM Tris-HCl pH 7.5, 0.1 M NaCl, 1% IGEPAL CA-630, 5 mM $MgCl_2$, 0.5% sodium deoxycholate, 0.1% SDS
**FA2**	50 mM HEPES-KOH pH 7.6, 500 mM NaCl, 1 mM EDTA, 1% Triton X-100, 0.1% Na-Deoxycholate
**FA3**	10 mM Tris-HCl pH 7.8, 250 mM $LiCl_2$, 1 mM EDTA, 0.5% IGEPAL CA-630, 0.5% Na-Deoxycholate
**WB1**	50 mM Tris-HCl pH 7.5, 500 mM NaCl, 0.1% IGEPAL CA-630, 10 mM imidazole, 6 M GuHCl
**WB2**	50 mM Tris-HCl pH 7.5, 500 mM NaCl, 0.1% Triton X-100
**WB3**	50 mM Tris-HCl pH 7.5, 500 mM NaCl, 0.1% Triton X-100, 8 M urea
**EB**	WB1 supplemented with 300 mM imidazole
**C**	50 mM Tris-HCl pH 7.8, 50 mM NaCl, 0.1% IGEPAL CA-630
**10x R**	700 mM Tris-HCl pH 7.5, 100 mM $MgCl_2$
**10x R2**	700 mM Bis-Tris pH 6.5, 100 mM $MgCl_2$
**PKB**	Buffer C supplemented with 5 mM EDTA and 1% SDS
**PCI**	25:24:1 mix of phenol pH 8.0, chloroform and isoamyl alcohol

Supplementary References

1. Goldfarb, K.C. & Cech, T.R. Targeted CRISPR disruption reveals a role for RNase MRP RNA in human preribosomal RNA processing. *Genes Dev* **31**, 59-71 (2017).
2. Esakova, O. & Krasilnikov, A.S. Of proteins and RNA: the RNase P/MRP family. *RNA* **16**, 1725-47 (2010).
3. Lemieux, B. et al. Active Yeast Telomerase Shares Subunits with Ribonucleoproteins RNase P and RNase MRP. *Cell* **165**, 1171-1181 (2016).
4. Busan, S. & Weeks, K.M. Accurate detection of chemical modifications in RNA by mutational profiling (MaP) with ShapeMapper 2. *Rna* **24**, 143-148 (2018).
5. Sloan, K.E. et al. Both endonucleolytic and exonucleolytic cleavage mediate ITS1 removal during human ribosomal RNA processing. *J Cell Biol* **200**, 577-88 (2013).
6. Wang, M., Anikin, L. & Pestov, D.G. Two orthogonal cleavages separate subunit RNAs in mouse ribosome biogenesis. *Nucleic Acids Res* **42**, 11180-91 (2014).
