# Supplementary material for: A disease-linked lncRNA mutation in RNase MRP inhibits ribosome synthesis
Source: Nat Commun. 2022 Feb 3;13:649. doi: 10.1038/s41467-022-28295-8 (PMC8814244; doi:10.1038/s41467-022-28295-8)
Supplement: Supplementary file 3 — Reporting Summary [file 41467_2022_28295_MOESM3_ESM.pdf]

nature research

Corresponding author(s): David Tollervey

Last updated by author(s): Apr 30, 2020

Reporting Summary

Nature Research wishes to improve the reproducibility of the work that we publish. This form provides structure for consistency and transparency in reporting. For further information on Nature Research policies, see our Editorial Policies and the Editorial Policy Checklist.

Statistics

For all statistical analyses, confirm that the following items are present in the figure legend, table legend, main text, or Methods section.

n/a	Confirmed	
☐	☒	The exact sample size (n) for each experimental group/condition, given as a discrete number and unit of measurement
☐	☒	A statement on whether measurements were taken from distinct samples or whether the same sample was measured repeatedly
☐	☒	The statistical test(s) used AND whether they are one- or two-sided<br>*Only common tests should be described solely by name; describe more complex techniques in the Methods section.*
☒	☐	A description of all covariates tested
☒	☐	A description of any assumptions or corrections, such as tests of normality and adjustment for multiple comparisons
☐	☒	A full description of the statistical parameters including central tendency (e.g. means) or other basic estimates (e.g. regression coefficient) AND variation (e.g. standard deviation) or associated estimates of uncertainty (e.g. confidence intervals)
☐	☒	For null hypothesis testing, the test statistic (e.g. F, t, r) with confidence intervals, effect sizes, degrees of freedom and P value noted<br>*Give P values as exact values whenever suitable.*
☒	☐	For Bayesian analysis, information on the choice of priors and Markov chain Monte Carlo settings
☒	☐	For hierarchical and complex designs, identification of the appropriate level for tests and full reporting of outcomes
☒	☐	Estimates of effect sizes (e.g. Cohen's d, Pearson's r), indicating how they were calculated

Our web collection on statistics for biologists contains articles on many of the points above.

Software and code

Policy information about availability of computer code

Data collection	Flow cytometry data was acquired with BD FACSDiva Software, version 8.0.1.<br>Incucyte software version: IncuCyte2011A.
Data analysis	Northern Blot images were analysed using ImageJ, version 1.51<br>Mass spectrometry data were analysed using MaxQuant software platform, version 1.6.1.<br>Flow cytometry data were analysed using FlowJo, version 10.6.<br>Graphs with associated statistics were prepared using Prism, version 9.0.0.<br>LightCycler software (version 1.5)

For manuscripts utilizing custom algorithms or software that are central to the research but not yet described in published literature, software must be made available to editors and reviewers. We strongly encourage code deposition in a community repository (e.g. GitHub). See the Nature Research guidelines for submitting code & software for further information.

April 2020

Data

Policy information about availability of data

All manuscripts must include a data availability statement. This statement should provide the following information, where applicable:

- Accession codes, unique identifiers, or web links for publicly available datasets
- A list of figures that have associated raw data
- A description of any restrictions on data availability

The GEO accession number for all sequence data reported in this paper is GSE171021. The proteomics data are available through the ProteomeXchange partner repository with the dataset identifier PXD025029. Raw data for all figures is provided.

R

Field-specific reporting

Please select the one below that is the best fit for your research. If you are not sure, read the appropriate sections before making your selection.

[x] Life sciences [ ] Behavioural & social sciences [ ] Ecological, evolutionary & environmental sciences

For a reference copy of the document with all sections, see nature.com/documents/nr-reporting-summary-flat.pdf

Life sciences study design

All studies must disclose on these points even when the disclosure is negative.

Sample size	Sample sizes were determined by technical restrictions of the experiments, and experiments were replicated to ensure statistical validity.
Data exclusions	No data was excluded from analysis, unless experimental control samples indicated technical deficiency of the experiment.
Replication	All experiments were performed at least twice, with most experiments including at least two technical replicates. Most key experiments were performed at least three times, as indicated in figure legends. One replicate of the TRAPP experiment was discarded as it failed to enrich for RNA binding proteins, probably due to incorrect measurement of initial nucleic acid concentration causing indiscriminate protein precipitation on the column. A subsequent repeat was successful. For the SHAPE-MaP experiments, one treated/untreated sample pair failed to meet the QC requirements for the SHAPE-Mapper software. This pair is not included in the analysis although the data is available on GEO.
Randomization	Samples were not randomised. For all experiments, covariants were controlled by comparing samples which were genetically identical other than the mutation or genetic perturbation of interest, and processing these in parallel. For mice experiments, cells from all animals in the experiment were pooled and then this pool split evenly between experimental samples or controls.
Blinding	The investigator was not blinded for experiments, due to practical requirements of experimental procedures. For mouse T cell CRISPR experiments, the order of sample electroporation (negative control and guide samples) was varied between experiments to reduce the chance of technical artifacts.

Reporting for specific materials, systems and methods

We require information from authors about some types of materials, experimental systems and methods used in many studies. Here, indicate whether each material, system or method listed is relevant to your study. If you are not sure if a list item applies to your research, read the appropriate section before selecting a response.

Materials & experimental systems

n/a	Involved in the study	
x		Antibodies
	x	Eukaryotic cell lines
x		Palaeontology and archaeology
	x	Animals and other organisms
x		Human research participants
x		Clinical data
x		Dual use research of concern

Methods

n/a	Involved in the study	
x		ChIP-seq
	x	Flow cytometry
x		MRI-based neuroimaging

Eukaryotic cell lines

Policy information about cell lines

Cell line source(s)	K562 cells were obtained from ATCC (cat. CCL-243). Patient and control fibroblasts were sourced from the Great North Biobank (REC number 16-NE-0002).
Authentication	Cell lines were not authenticated but were obtained from verified sources at the start of the project.
Mycoplasma contamination	Cell lines were not tested for mycoplasma contamination.
Commonly misidentified lines (See ICLAC register)	No commonly misidentified cell lines were used.

Animals and other organisms

Policy information about studies involving animals; ARRIVE guidelines recommended for reporting animal research

Laboratory animals	All mice used were Rag1 KO, C57BL6/J animals which were homozygous for the OT-1 allele. All animals were aged between 2 and months at the time of experiment. Both male and female animals were used. Cells from all animals in the experiment were pooled and then split evenly between experimental samples. Animal facilities were maintained at a temperature of 21°C, and a humidity of 50 - 60%. Light was varied on a 12 hour cycle (dark from 7 pm to 7 am, and light from 7 am to 7 pm).
Wild animals	No wild animals used.
Field-collected samples	No field-collected samples were used.
Ethics oversight	All experimental procedures were approved by a current project license under the authority of the Animals (Scientific Procedures) Act 1986, and additionally followed the University of Edinburgh's ethical guidance as overseen by its AWERB committee.

Note that full information on the approval of the study protocol must also be provided in the manuscript.

Flow Cytometry

Plots

Confirm that:

- [x] The axis labels state the marker and fluorochrome used (e.g. CD4-FITC).
- [x] The axis scales are clearly visible. Include numbers along axes only for bottom left plot of group (a 'group' is an analysis of identical markers).
- [x] All plots are contour plots with outliers or pseudocolor plots.
- [x] A numerical value for number of cells or percentage (with statistics) is provided.

Methodology

Sample preparation	For mouse experiments: Peripheral lymph nodes were dissected from Rag1 knockout mice homozygous for the OT1 allele. Lymph nodes were massaged through a 70 µM cell strainer. Cells were washed once with IMDM (Gibco; cat. 12440053) supplemented with 2mM L-glutamine, counted, and resuspended at 5 x 106 cells/mL in PBS supplemented with 2.5 µM CellTrace Violet (Invitrogen; cat. C34557). After 20 minutes at 37 oC, cells were washed with complete T Cell media (TCM; IMDM supplemented with 10% FCS, 2 mM L-glutamine, 100 U/mL penicillin, 100 U/mL streptomycin, and 50 µM 2-mercaptoethanol), counted, and resuspended at 250,000 cells/mL in TCM supplemented with 10 nM N4 peptide (peptide sequence SIINFEKL). After 24 hours of stimulation, cells were counted, pelleted, and resuspended for transfection at 1.5 million cells per transfection in 80 µL Neon Transfection System Buffer T (Invitrogen; cat. MPK10096). CRISPR guide RNAs were purchased from IDT (Table 1), and resuspended at 100 µM in Nuclease-Free Duplex Buffer (IDT; cat. 11-01-03-01). For each transfection, 2.5 µL of guide RNA was mixed with 2.5 µL of tracrRNA and 20 µL of Nuclease-Free Duplex Buffer. The mix was heated at 95 oC for 5 minutes and then allowed to cool. Once at room temperature, 23 µL of Buffer T and 2 µL of TrueCut Cas9 Protein v2 (5 mg/mL stock; Invitrogen; cat A36496) was added, and the combined mix heated to 37 oC for 10 minutes before electroporation using a Neon Transfection System with 3x 1600 V pulses of width 10 ms. Electroporated cells were then cultured for 48 hours at either 250,000 (for flow cytometry) or 1 x 106 cells per ml (for Northern blotting) in TCM supplemented with recombinant IL-2 (20 ng/mL). For flow cytometry, cells were first washed once with PBS before live/dead staining with Zombie Red Fixable Viability Kit (1:100 in PBS for 20 mins at room temperature; BioLegend; cat. 423109). Cells were then washed twice with FACS buffer before sample acquisition.<br>For FlowFISH experiments: 0.5 x 106 K562 cells were used. Cells were pelleted at 500 g for 5 minutes at room temperature, and washed once with 0.5 mL of PBS. After pelleting, cells were resuspended in 0.5 mL of PBS, and 0.5 mL of 8% paraformaldehyde added. Cells were left to fix for 30 minutes at room temperature, after which they were washed twice with 1 mL of PBS and resuspended in 0.5 mL PBS. 0.5 mL of 70% ethanol was then added dropwise, and cells pelleted again before resuspension in 1 mL of 70% ethanol and permeabilization overnight at 4oC. FlowFISH wash buffer (FFWB) was prepared by supplementing 2x SSC with 10% formamide and 0.25 mg/mL Bovine Serum Albumin fraction V (BSA; Sigma; cat. 05482). FlowFISH solution A (FFSA) was prepared by combining, per sample: 5 µL of formamide; 2.5 µL of 2x SSC; 2.5 µL of 10 mg/mL E. coli tRNA (Sigma; cat. R1753); 2.5 µL of FISH probes diluted to 50 ng/µL; and 8.75 µL of MQ. FFSA was then heated to 95 oC and allowed to cool. Meanwhile, FlowFISH solution B (FFSB) was prepared by combining, per sample: 25 µL of 20%

dextran sulphate dissolved in 4x SSC; 1.25 µL of 10 mg / mL BSA; and 40 units of RNasin Ribonuclease Inhibitor (Promega; cat. N211A). Once FFSA was cool, FFSA and FFSB were combined 1:1 to create the staining mix.

For staining, cells in ethanol were pelleted at 1000 g for 5 minutes, resuspended in 1 mL of FFWB and left to rehydrate at room temperature for 5 minutes before again being pelleted. Cells were then resuspended in staining mix for 3 hours at 37oC, before 2 washes with 1 mL FF wash buffer, and 2 washes with 1 mL FACS buffer.

Instrument

Samples were acquired on a custom research product BD LSRFortessa.

Software

Flow cytometry data were analysed using FlowJo, version 10.6.

Cell population abundance

Cells of interest made up >70% of total events, as indicated in Fig. S2.

Gating strategy

In mouse T cell experiments, cells were gated first on morphological lymphocytes (Fig. S2A), then live cells as assessed by ZombieRed live-dead stain on the APC channel. In FlowFish experiments, K562 cells were gated first on morphologically live cells (S2B) then on single cells.

☒ Tick this box to confirm that a figure exemplifying the gating strategy is provided in the Supplementary Information.
