# Supplementary material for: A disease-linked lncRNA mutation in RNase MRP inhibits ribosome synthesis
Source: Nat Commun. 2022 Feb 3;13:649. doi: 10.1038/s41467-022-28295-8 (PMC8814244; doi:10.1038/s41467-022-28295-8)
Supplement: Supplementary file 4 — Source Data [file 41467_2022_28295_MOESM4_ESM.zip › SourceData/NorthernBlots/RobertsonEtAl-2021-NorthernBlots/RobertsonEtAl-NorthernBlots.pptx]

## Slide 1
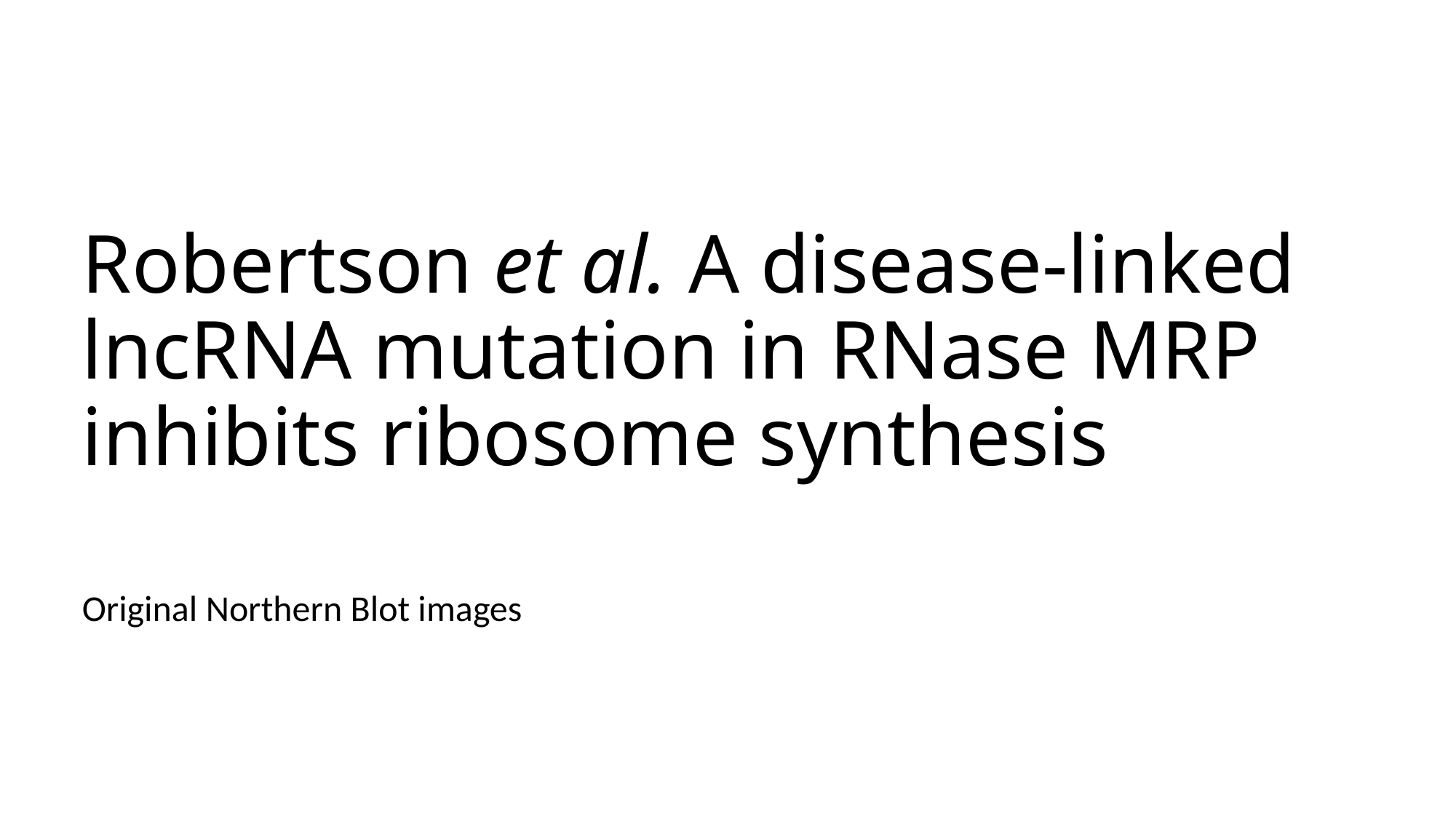

# Robertson et al. A disease-linked lncRNA mutation in RNase MRP inhibits ribosome synthesis
Original Northern Blot images

## Slide 2
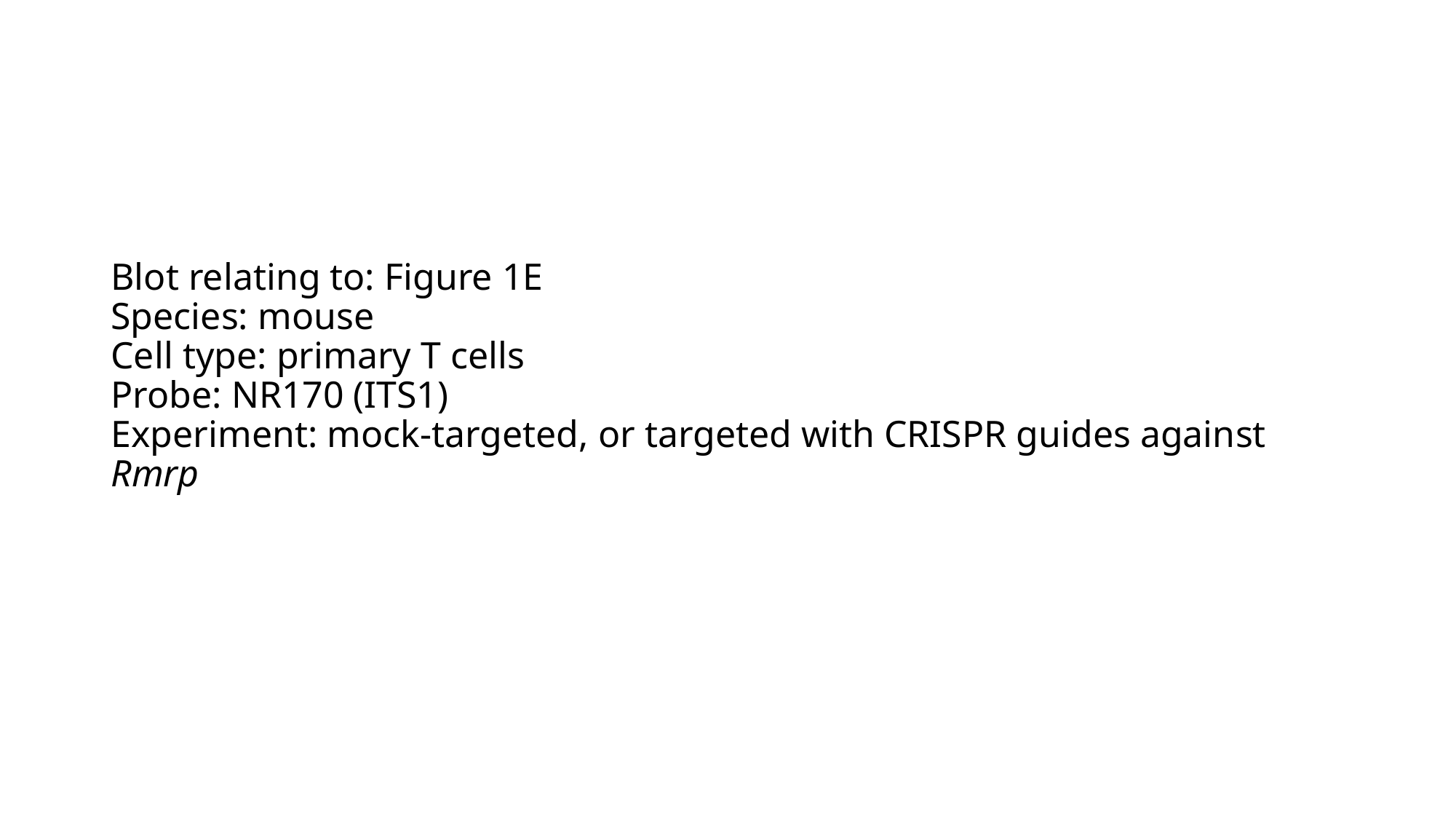

# Blot relating to: Figure 1E Species: mouse Cell type: primary T cells Probe: NR170 (ITS1) Experiment: mock-targeted, or targeted with CRISPR guides against Rmrp

## Slide 3
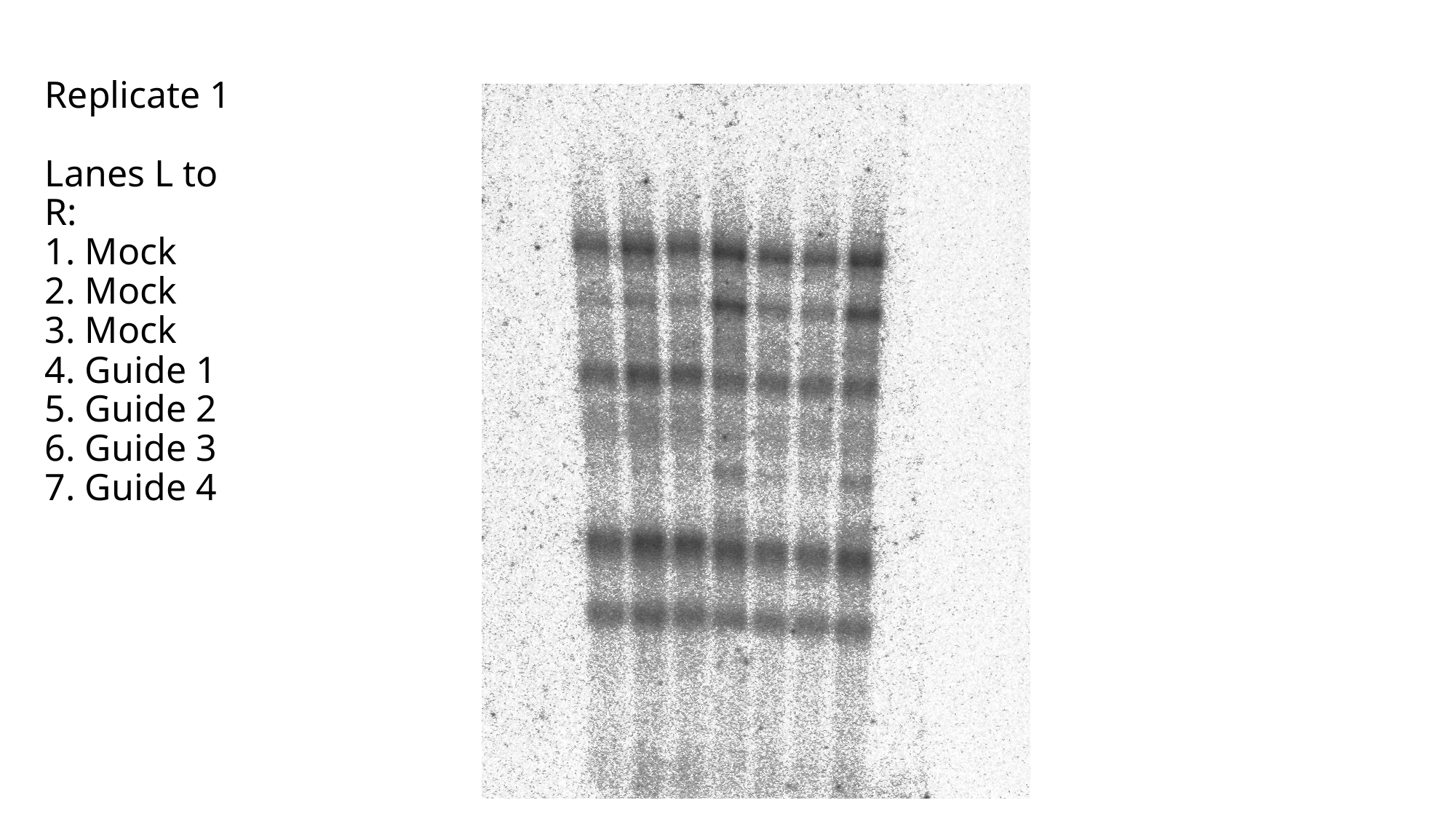

# Replicate 1 Lanes L to R: 1. Mock 2. Mock 3. Mock 4. Guide 1 5. Guide 2 6. Guide 3 7. Guide 4

## Slide 4
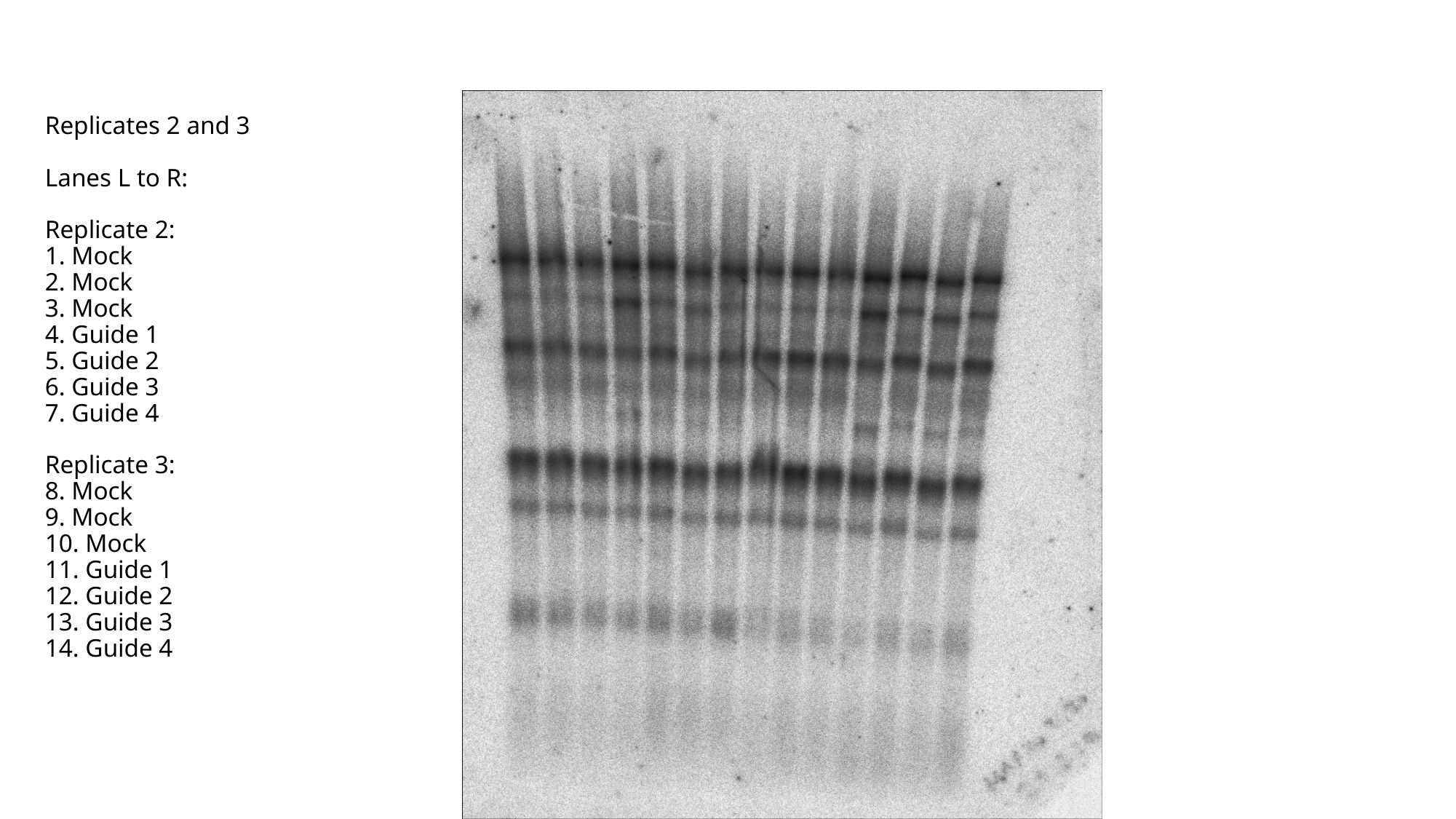

# Replicates 2 and 3 Lanes L to R: Replicate 2: 1. Mock 2. Mock 3. Mock 4. Guide 1 5. Guide 2 6. Guide 3 7. Guide 4 Replicate 3: 8. Mock 9. Mock 10. Mock 11. Guide 1 12. Guide 2 13. Guide 3 14. Guide 4

## Slide 5
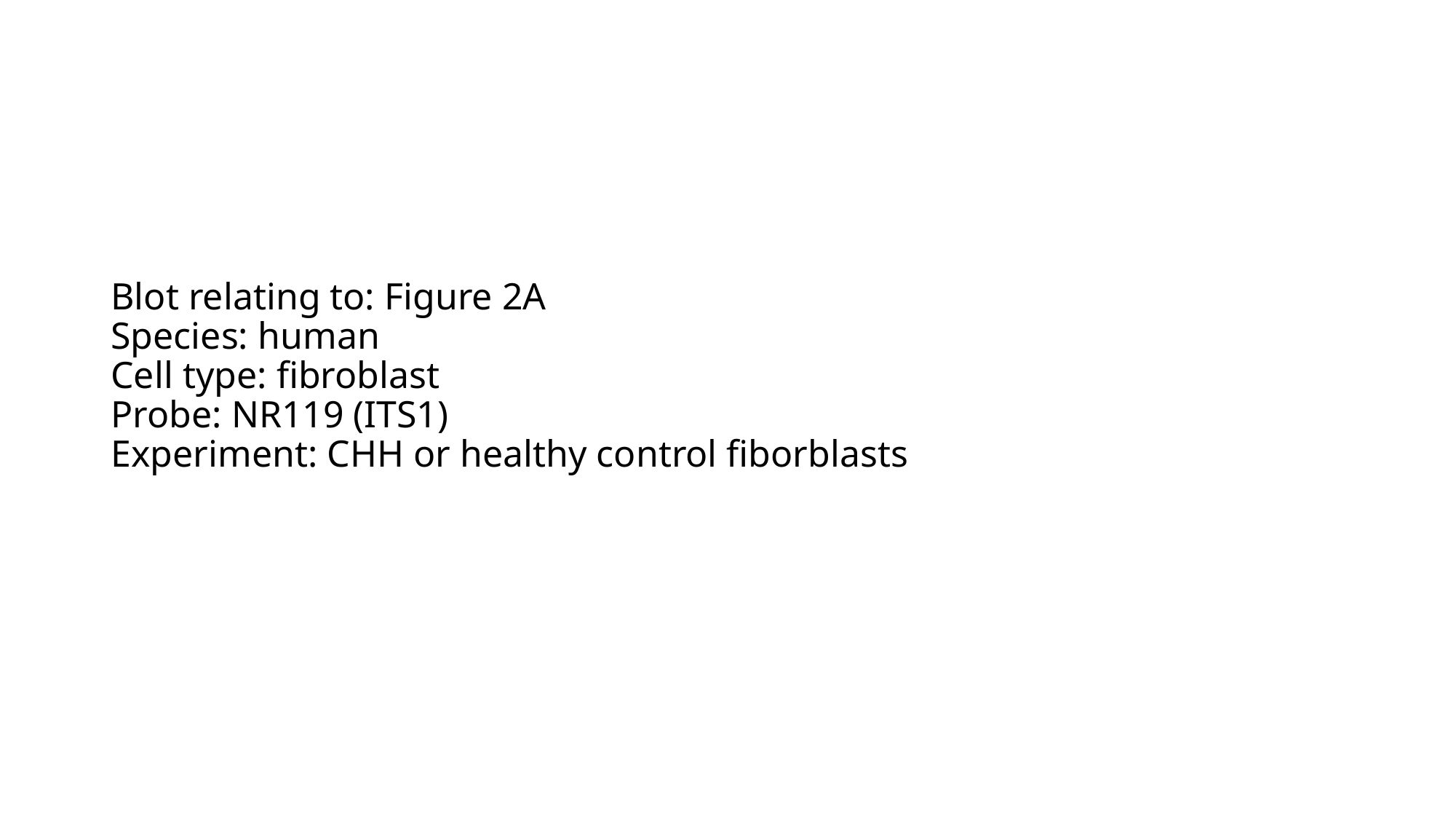

# Blot relating to: Figure 2A Species: human Cell type: fibroblast Probe: NR119 (ITS1) Experiment: CHH or healthy control fiborblasts

## Slide 6
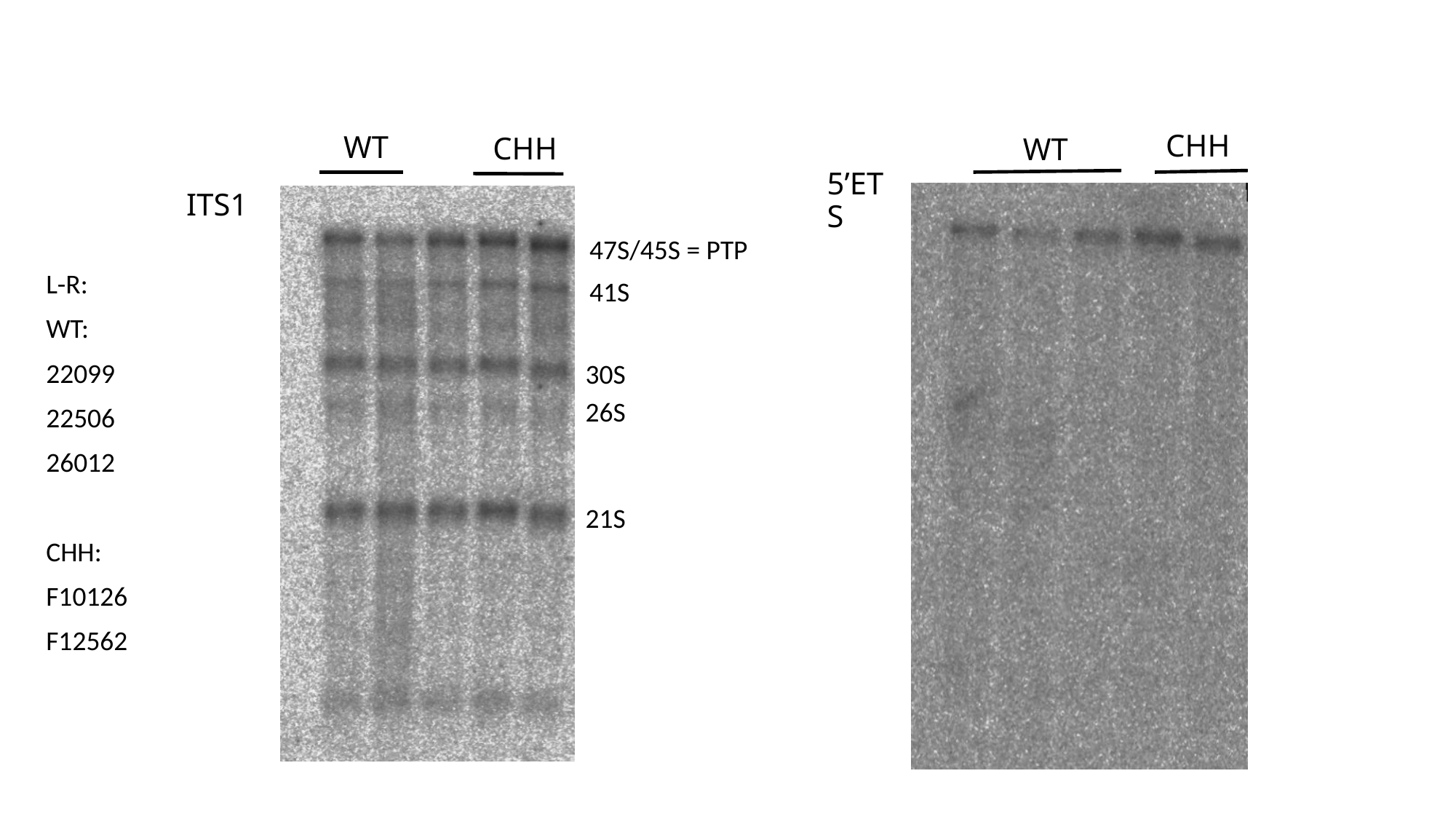

CHH
WT
CHH
WT
5’ETS
ITS1
47S/45S = PTP
L-R:
WT:
22099
22506
26012
CHH:
F10126
F12562
41S
30S
26S
21S

## Slide 7
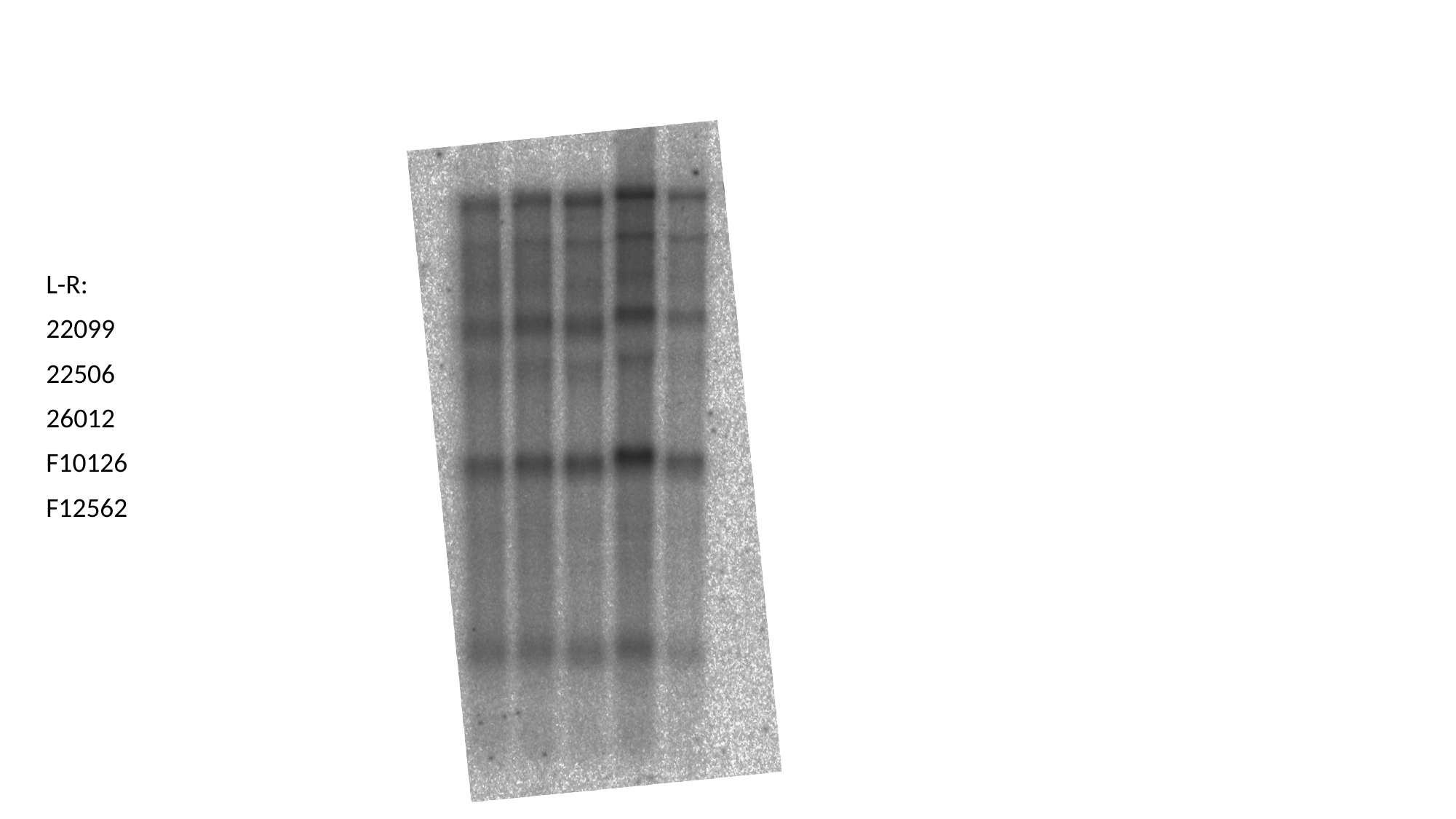

L-R:
22099
22506
26012
F10126
F12562

## Slide 8
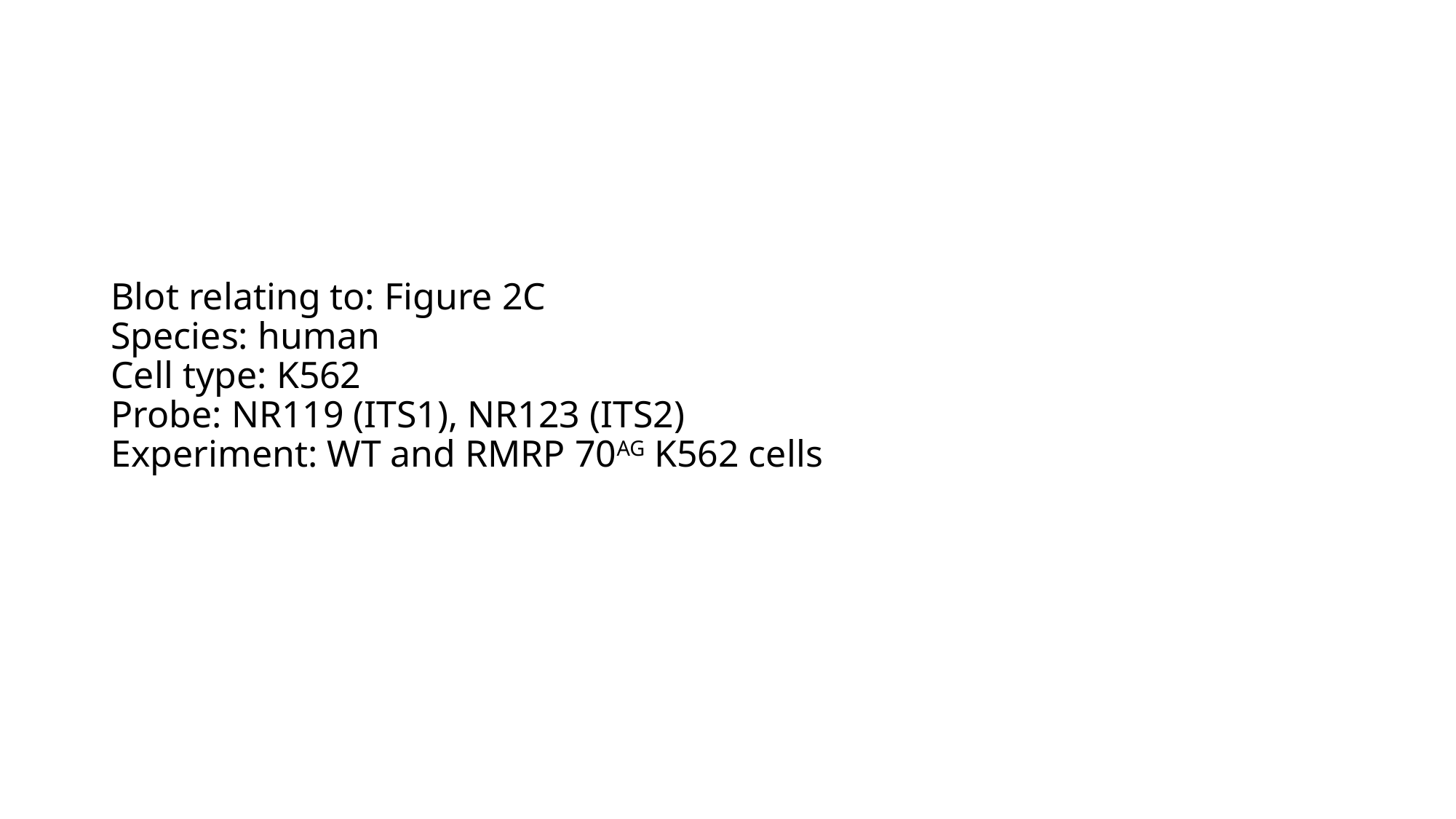

# Blot relating to: Figure 2C Species: human Cell type: K562 Probe: NR119 (ITS1), NR123 (ITS2) Experiment: WT and RMRP 70AG K562 cells

## Slide 9
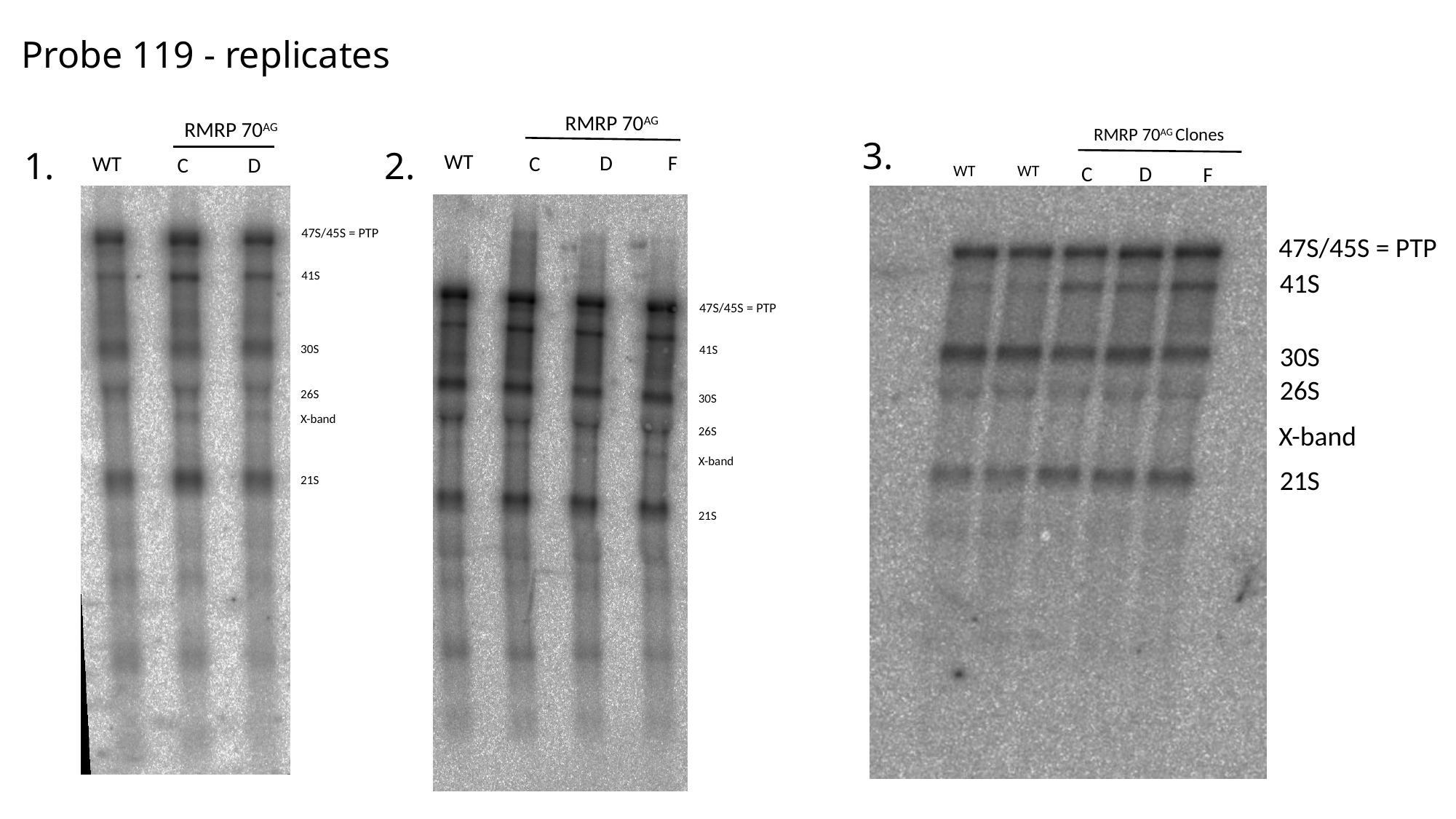

# Probe 119 - replicates
RMRP 70AG
3.
RMRP 70AG
1.
2.
RMRP 70AG Clones
WT
WT
D
C
F
47S/45S = PTP
41S
30S
26S
X-band
21S
WT
F
D
C
WT
D
C
47S/45S = PTP
41S
47S/45S = PTP
30S
41S
26S
30S
X-band
26S
X-band
21S
21S

## Slide 10
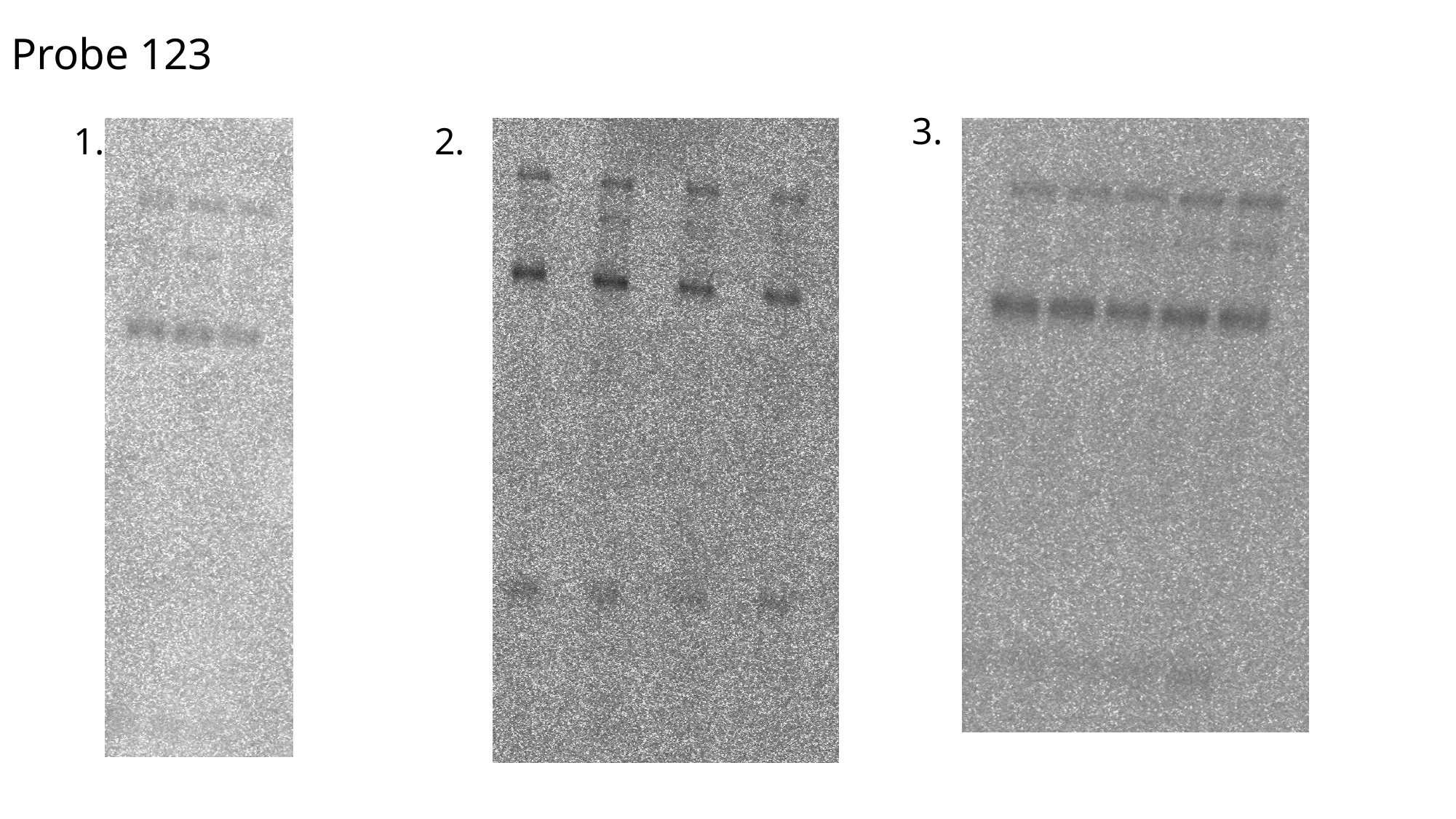

# Probe 123
3.
1.
2.

## Slide 11
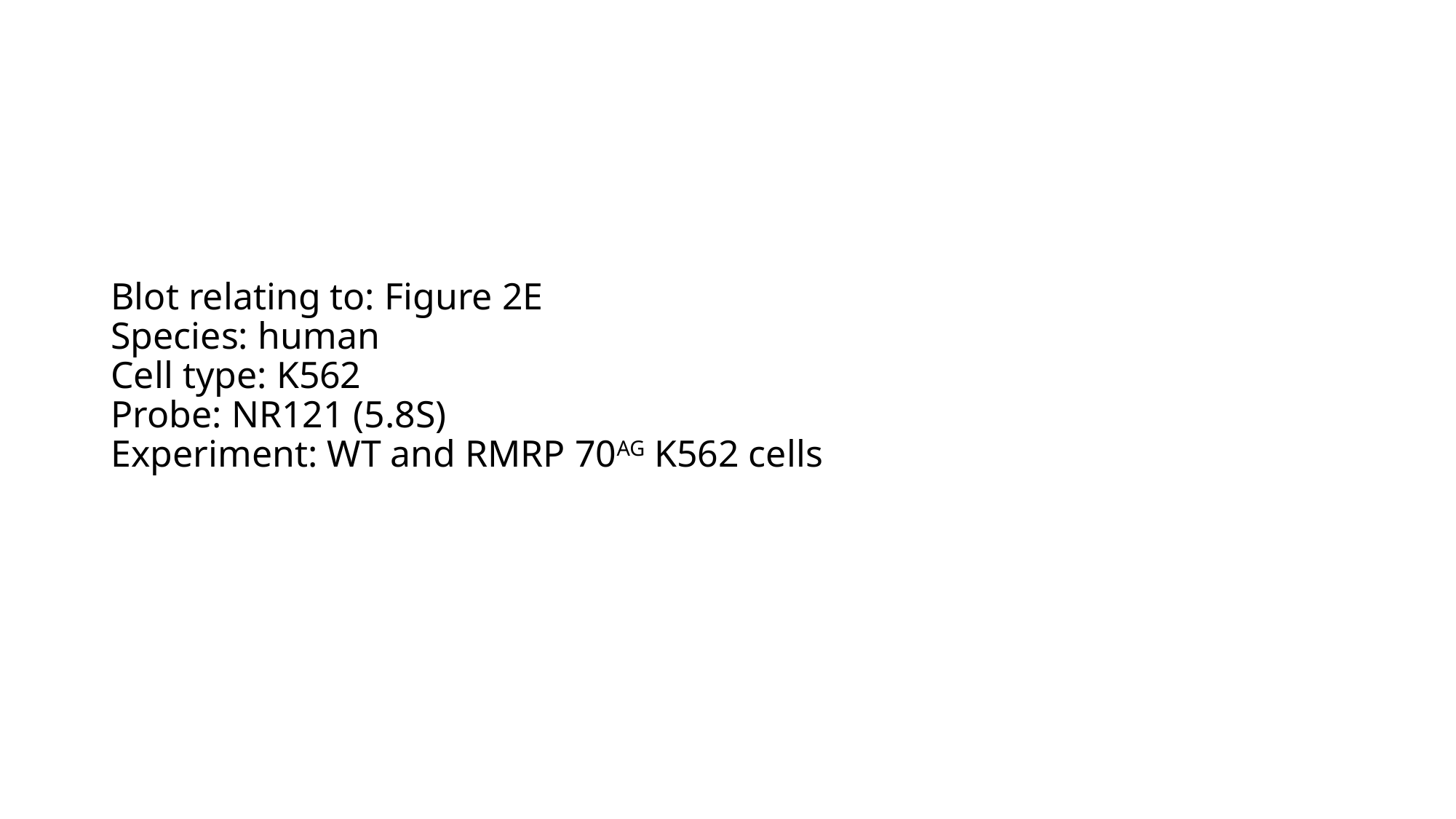

# Blot relating to: Figure 2E Species: human Cell type: K562 Probe: NR121 (5.8S) Experiment: WT and RMRP 70AG K562 cells

## Slide 12
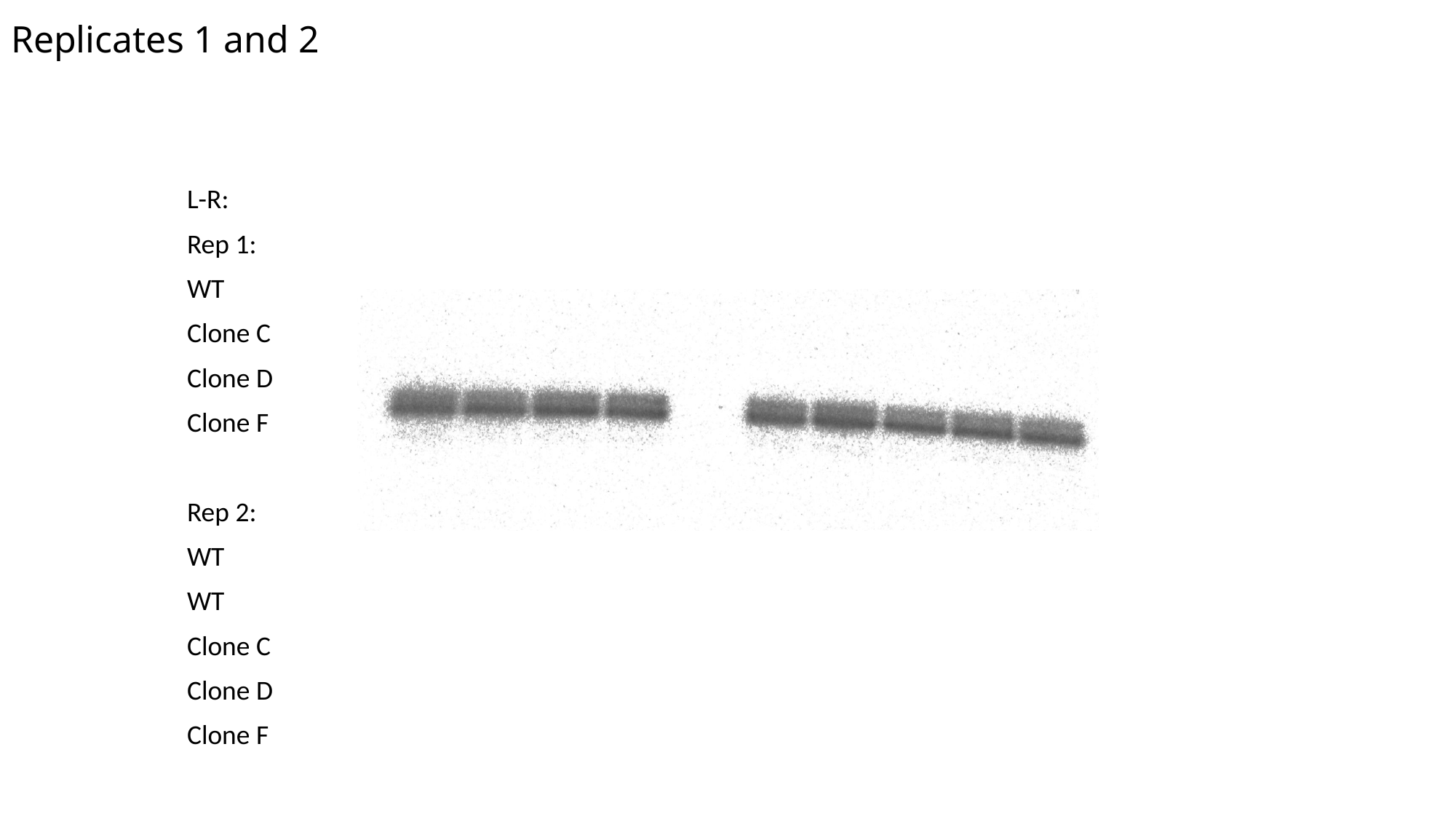

# Replicates 1 and 2
L-R:
Rep 1:
WT
Clone C
Clone D
Clone F
Rep 2:
WT
WT
Clone C
Clone D
Clone F

## Slide 13
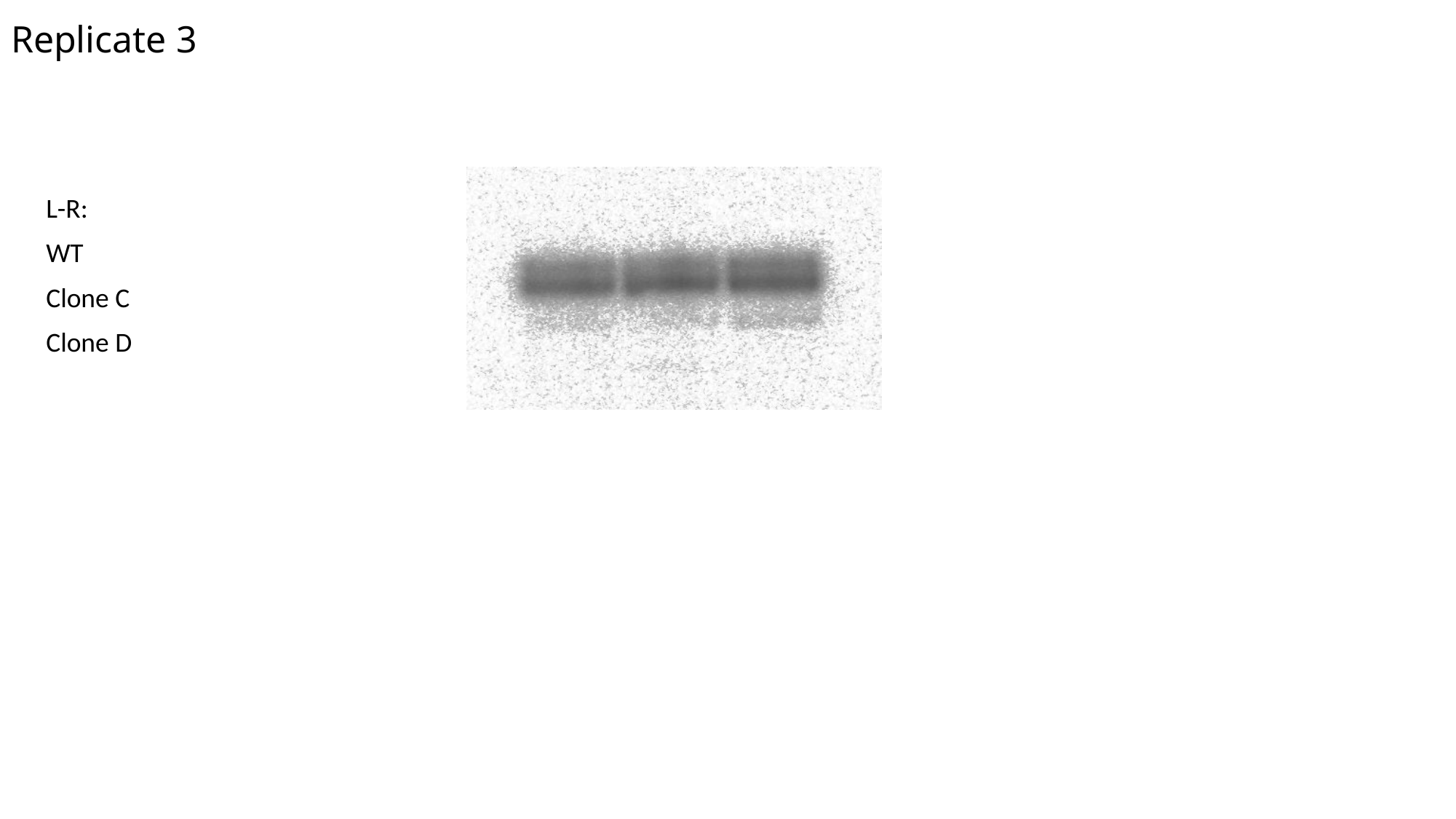

# Replicate 3
L-R:
WT
Clone C
Clone D
