# Supplementary material for: A disease-linked lncRNA mutation in RNase MRP inhibits ribosome synthesis
Source: Nat Commun. 2022 Feb 3;13:649. doi: 10.1038/s41467-022-28295-8 (PMC8814244; doi:10.1038/s41467-022-28295-8)

RNA: RMRP

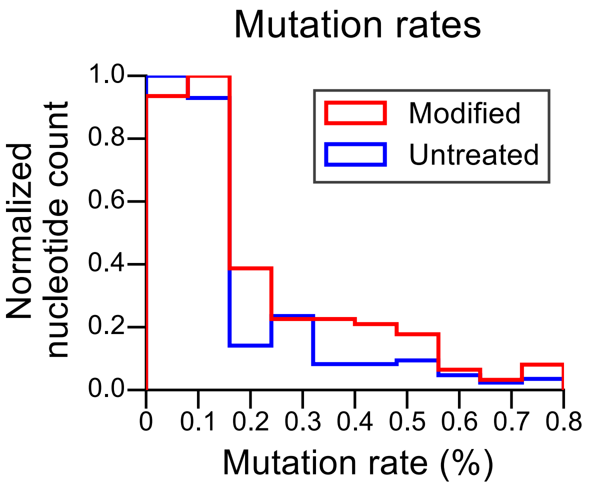

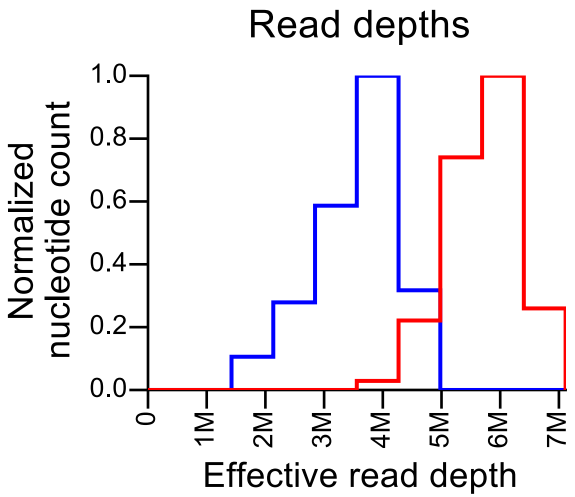

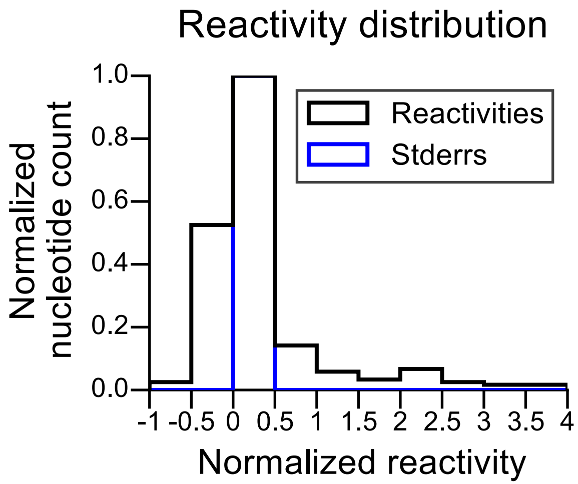

Modified sample:
95th percentile rate: 1.77%
Median rate: 0.16%

Untreated sample:
95th percentile rate: 0.78%
Median rate: 0.11%

Modified sample:
Median depth: 5,860,587
5th percentile depth: 4,581,556

Untreated sample:
Median depth: 3,693,367
5th percentile depth: 2,171,114

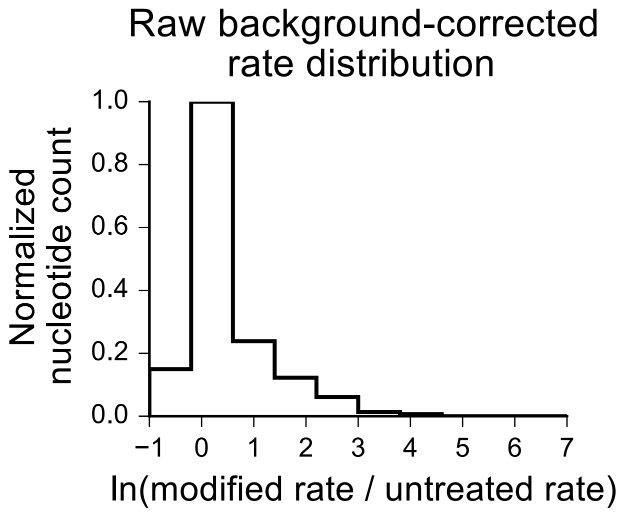

Supplement: Supplementary file 4 — Source Data [file 41467_2022_28295_MOESM4_ESM.zip › SourceData/RMRP-SHAPE-MaP/70AG_RMRP_3_RMRP_histograms.pdf]
