# Supplementary material for: A disease-linked lncRNA mutation in RNase MRP inhibits ribosome synthesis
Source: Nat Commun. 2022 Feb 3;13:649. doi: 10.1038/s41467-022-28295-8 (PMC8814244; doi:10.1038/s41467-022-28295-8)

RNA: RMRP Note: possible data quality issue - see log file

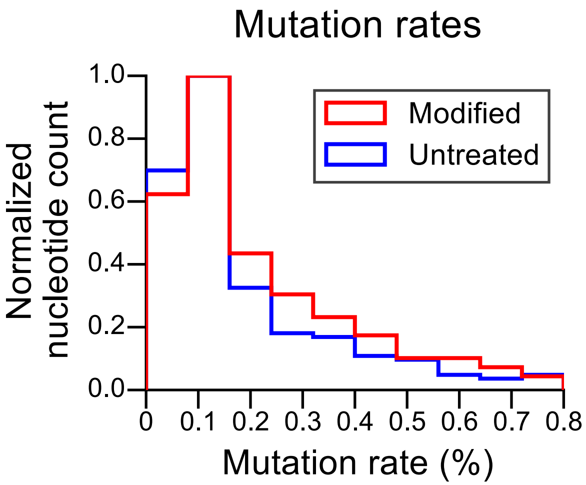

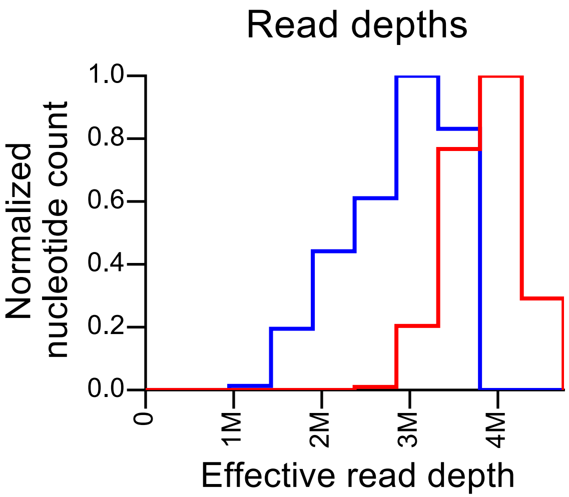

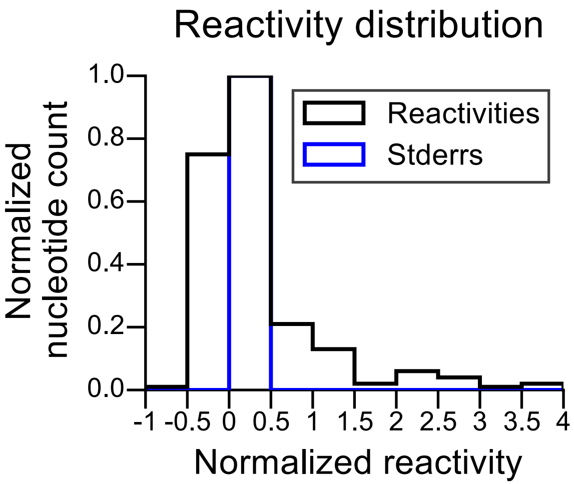

Modified sample:
95th percentile rate: 1.3%
Median rate: 0.17%

Untreated sample:
95th percentile rate: 0.81%
Median rate: 0.13%

Modified sample:
Median depth: 3,894,595
5th percentile depth: 3,150,506

Untreated sample:
Median depth: 2,977,791
5th percentile depth: 1,720,537

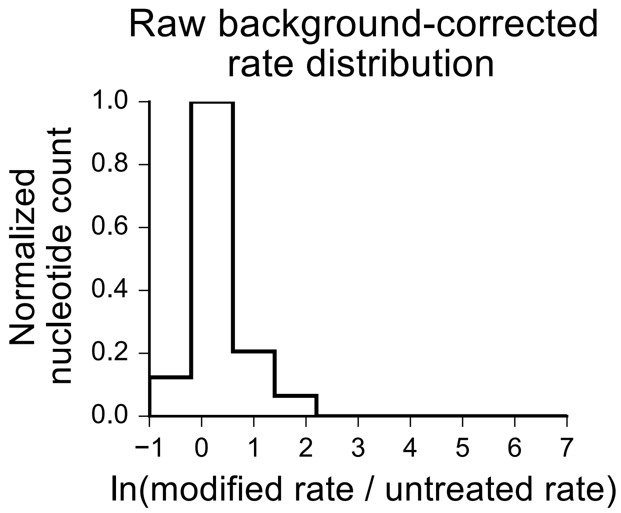

Supplement: Supplementary file 4 — Source Data [file 41467_2022_28295_MOESM4_ESM.zip › SourceData/RMRP-SHAPE-MaP/70AG_RMRP_2_RMRP_histograms.pdf]
