# Supplementary material for: A disease-linked lncRNA mutation in RNase MRP inhibits ribosome synthesis
Source: Nat Commun. 2022 Feb 3;13:649. doi: 10.1038/s41467-022-28295-8 (PMC8814244; doi:10.1038/s41467-022-28295-8)

RNA: RMRP

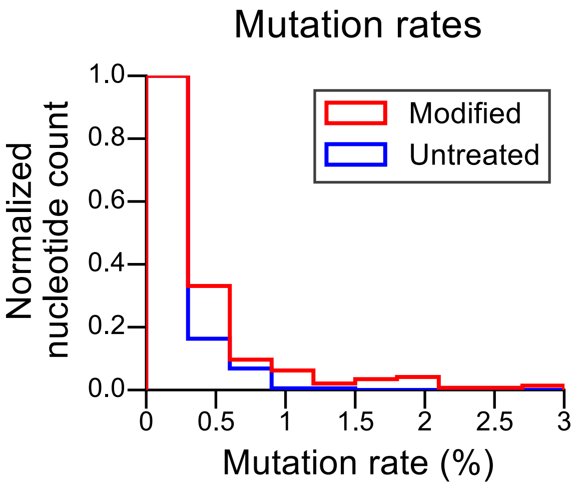

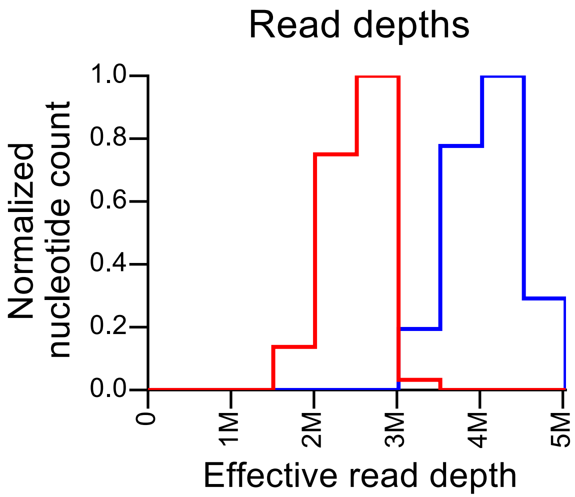

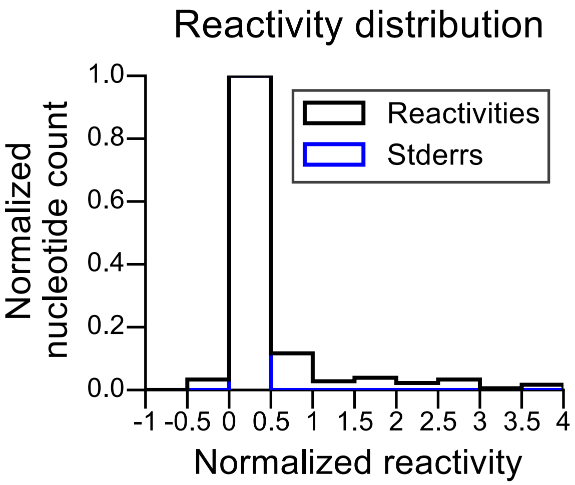

Modified sample:	
95th percentile rate:	1.9%
Median rate:	0.2%
Untreated sample:	
95th percentile rate:	0.7%
Median rate:	0.11%

Modified sample:	
Median depth:	2,550,873
5th percentile depth:	1,939,608
Untreated sample:	
Median depth:	4,114,873
5th percentile depth:	3,347,766

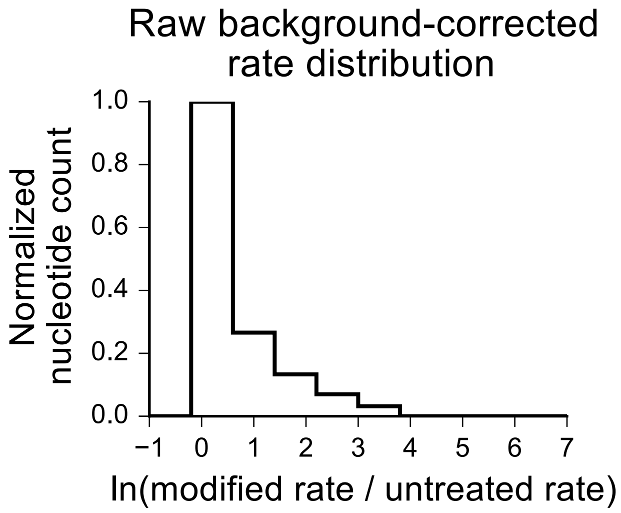

Supplement: Supplementary file 4 — Source Data [file 41467_2022_28295_MOESM4_ESM.zip › SourceData/RMRP-SHAPE-MaP/70AG_RMRP_1_RMRP_histograms.pdf]
