# Supplementary material for: A disease-linked lncRNA mutation in RNase MRP inhibits ribosome synthesis
Source: Nat Commun. 2022 Feb 3;13:649. doi: 10.1038/s41467-022-28295-8 (PMC8814244; doi:10.1038/s41467-022-28295-8)

RNA: RMRP

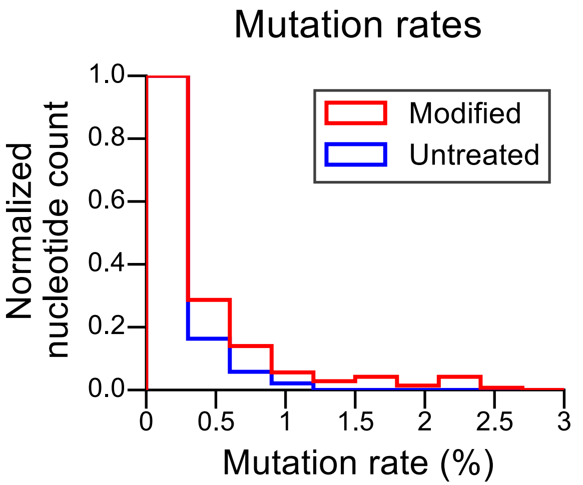

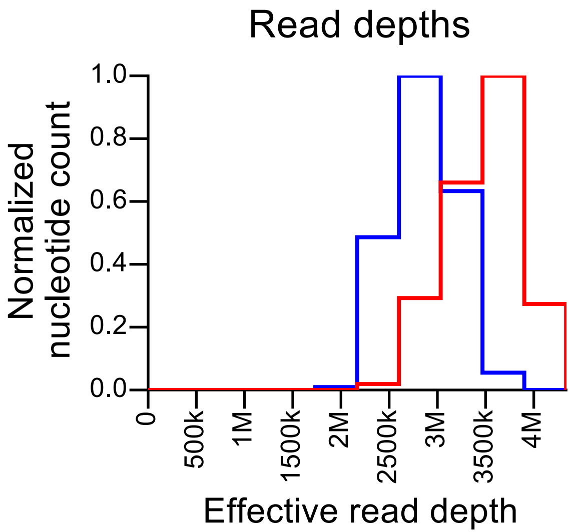

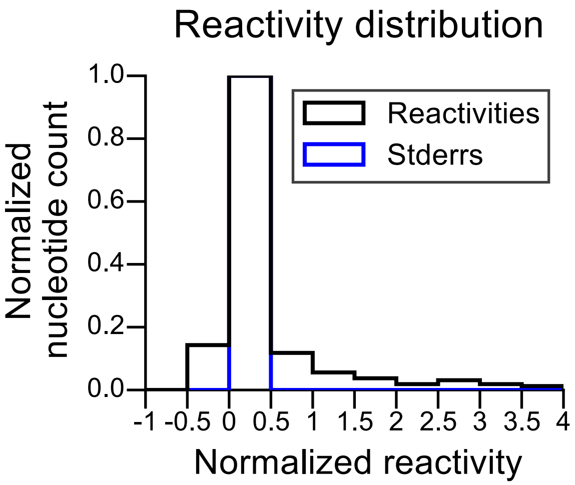

Modified sample:

95th percentile rate:	2.15%
Median rate:	0.2%

Untreated sample:

95th percentile rate:	0.69%
Median rate:	0.11%

Modified sample:

Median depth:	3,547,939
5th percentile depth:	2,842,642

Untreated sample:

Median depth:	2,917,896
5th percentile depth:	2,352,584

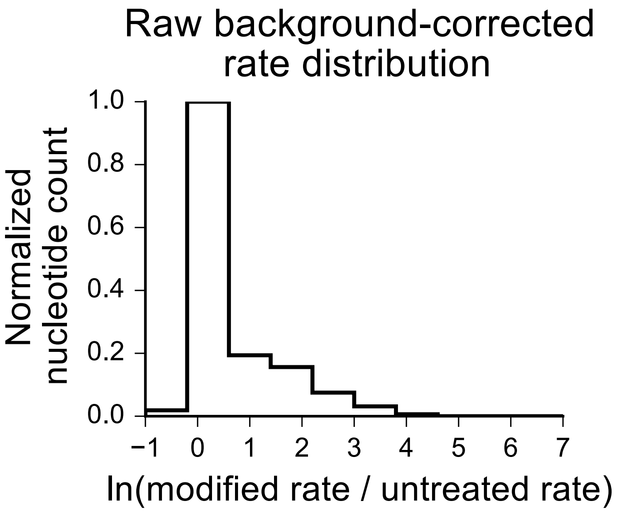

Supplement: Supplementary file 4 — Source Data [file 41467_2022_28295_MOESM4_ESM.zip › SourceData/RMRP-SHAPE-MaP/Wildtype_RMRP_1_RMRP_histograms.pdf]
