# Supplementary figures and images for: A disease-linked lncRNA mutation in RNase MRP inhibits ribosome synthesis
Source: Nat Commun. 2022 Feb 3;13:649. doi: 10.1038/s41467-022-28295-8 (PMC8814244; doi:10.1038/s41467-022-28295-8)

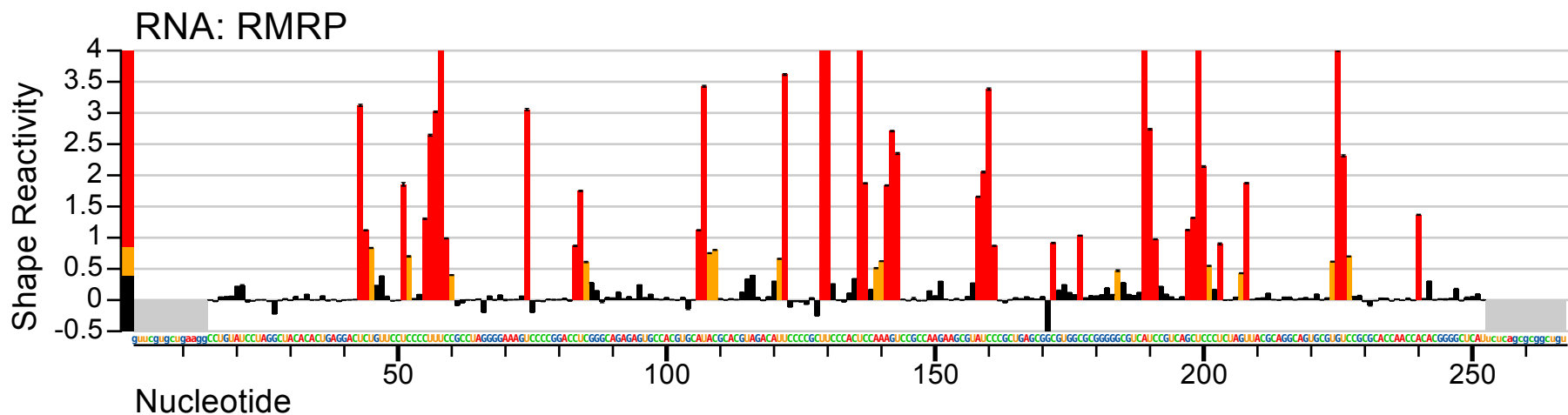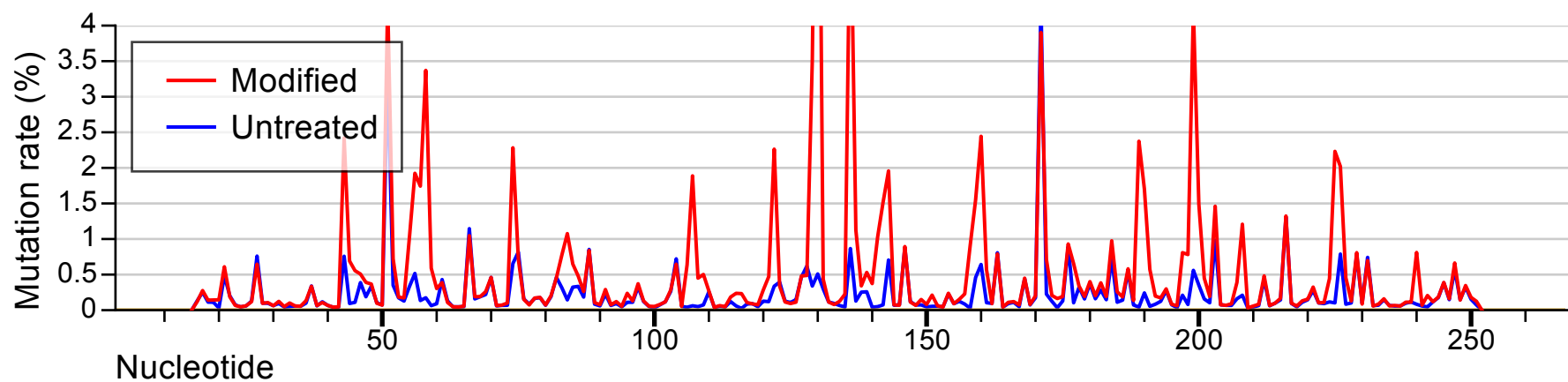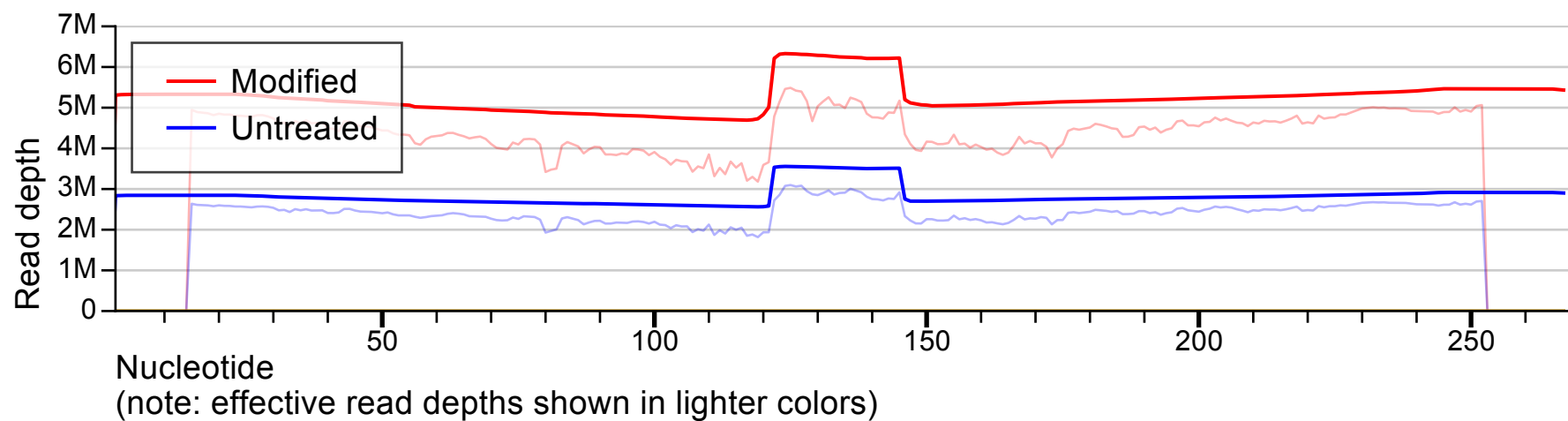

Supplement: Supplementary file 4 — Source Data [file 41467_2022_28295_MOESM4_ESM.zip › SourceData/RMRP-SHAPE-MaP/Wildtype_RMRP_3_RMRP_profiles.pdf]

$\Delta$ SHAPE

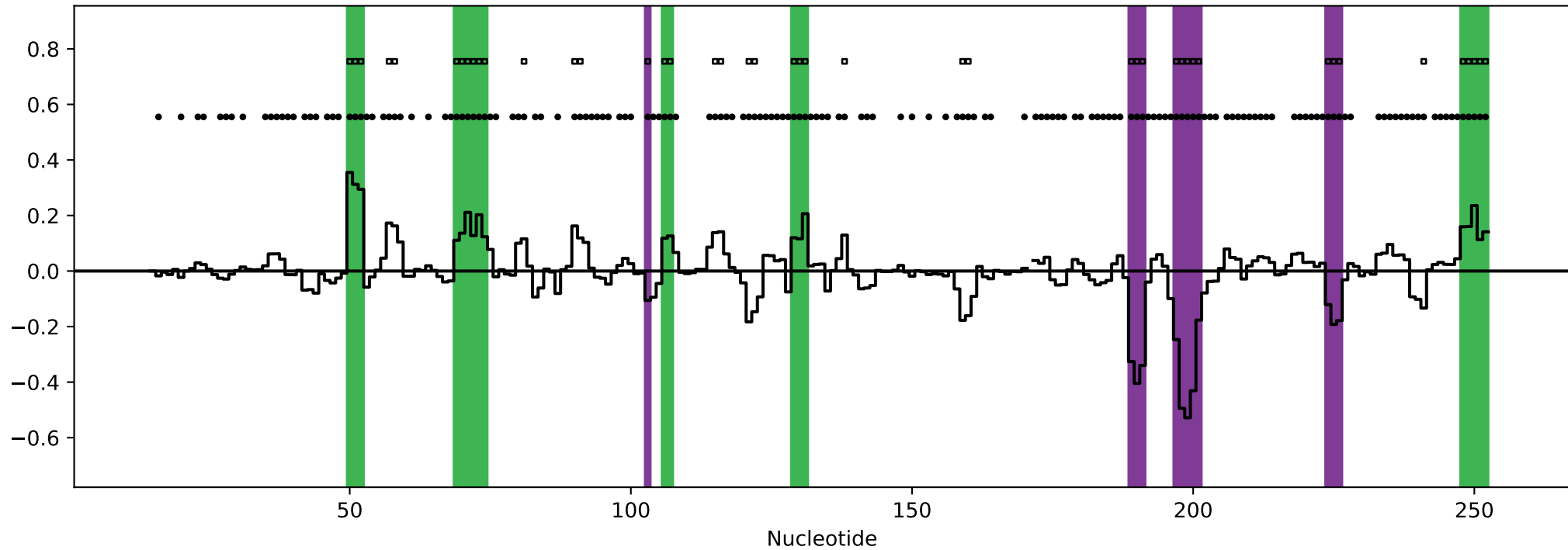

Supplement: Supplementary file 4 — Source Data [file 41467_2022_28295_MOESM4_ESM.zip › SourceData/RMRP-SHAPE-MaP/70AG1vsWT1.pdf]

# RNA: RMRP

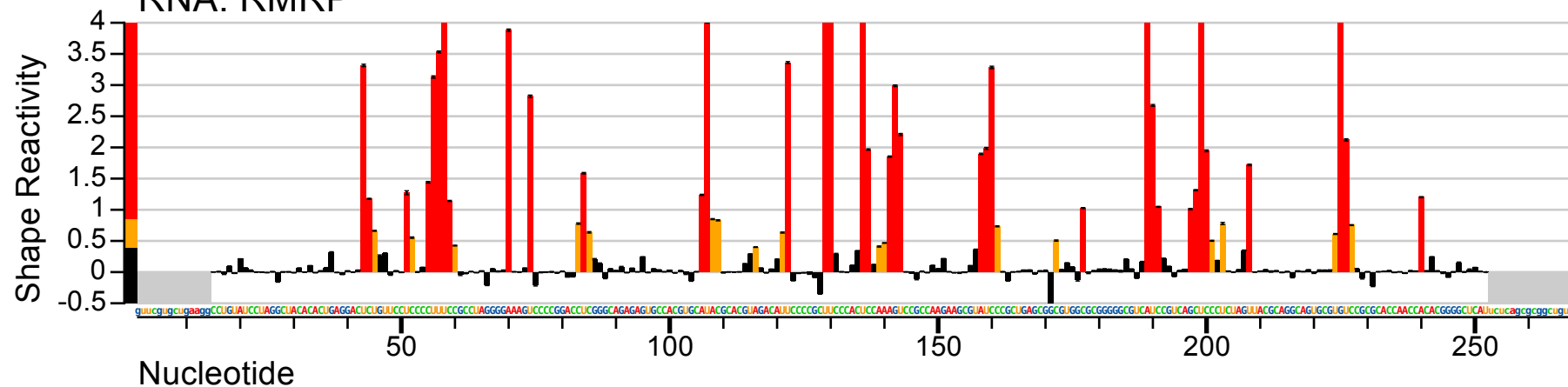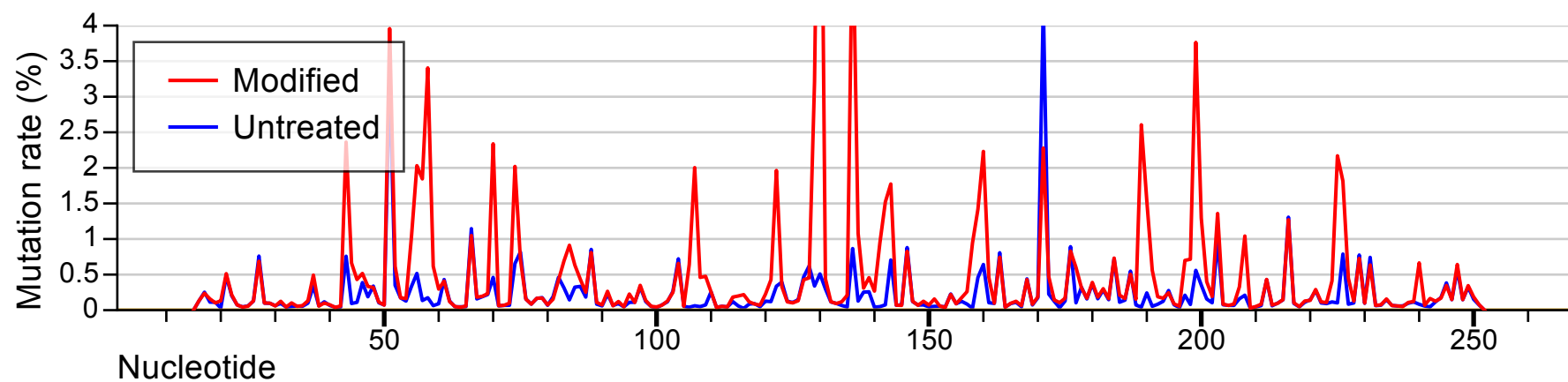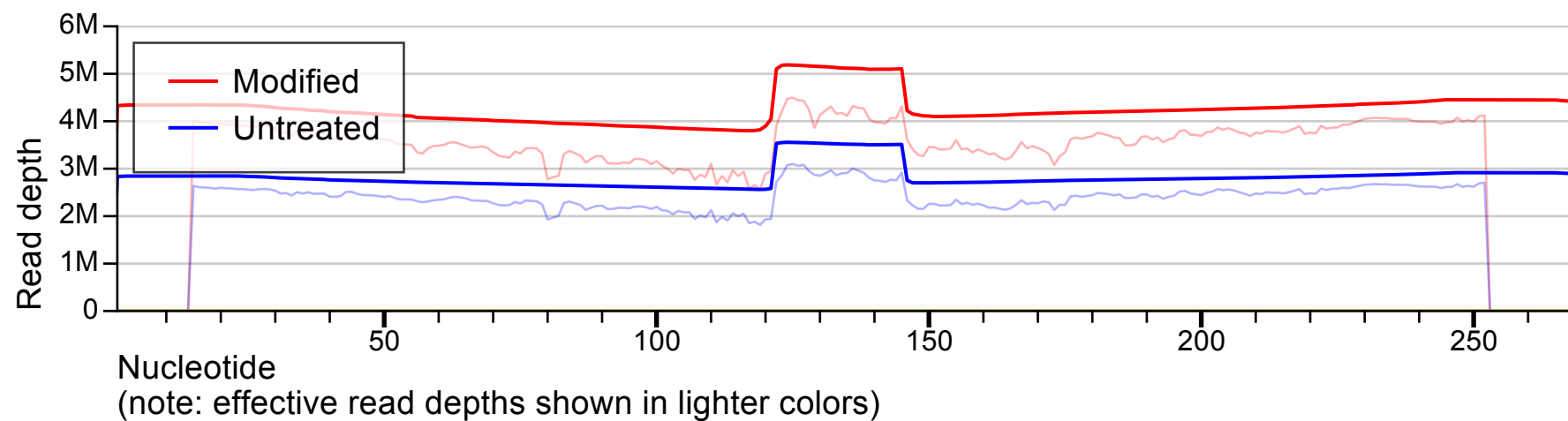

Supplement: Supplementary file 4 — Source Data [file 41467_2022_28295_MOESM4_ESM.zip › SourceData/RMRP-SHAPE-MaP/Wildtype_RMRP_2_RMRP_profiles.pdf]

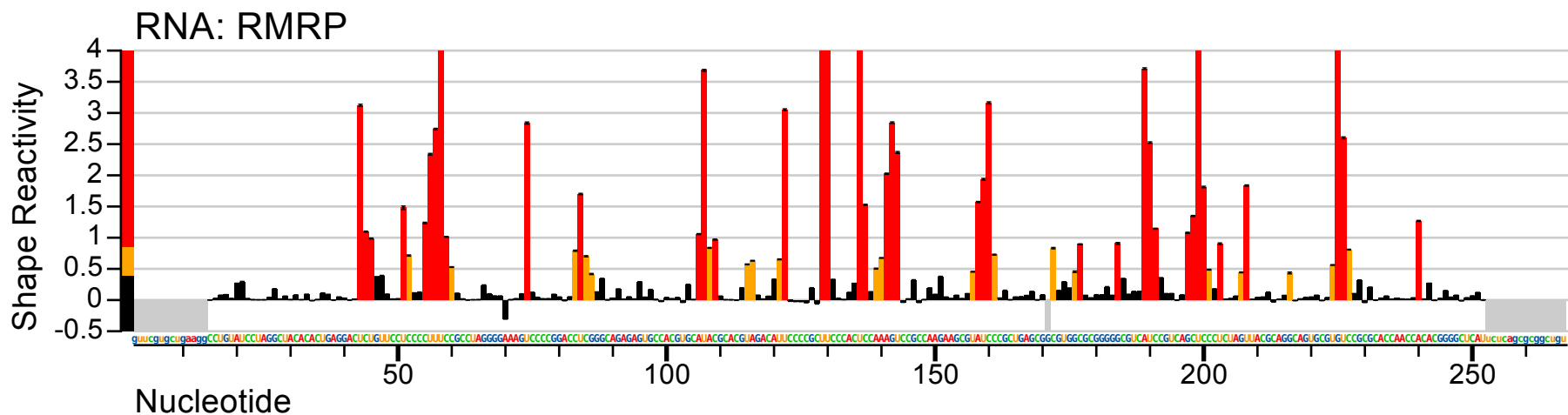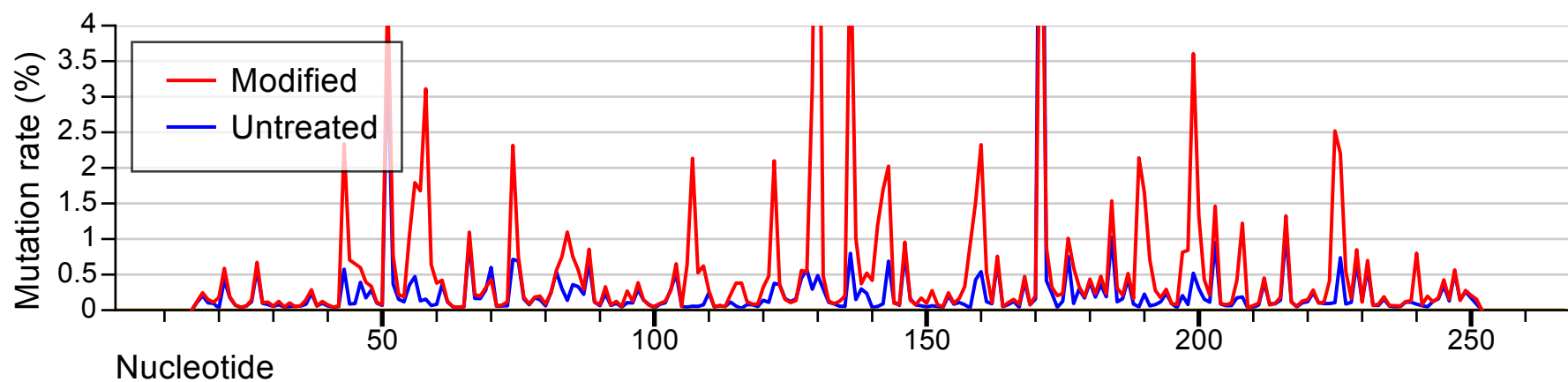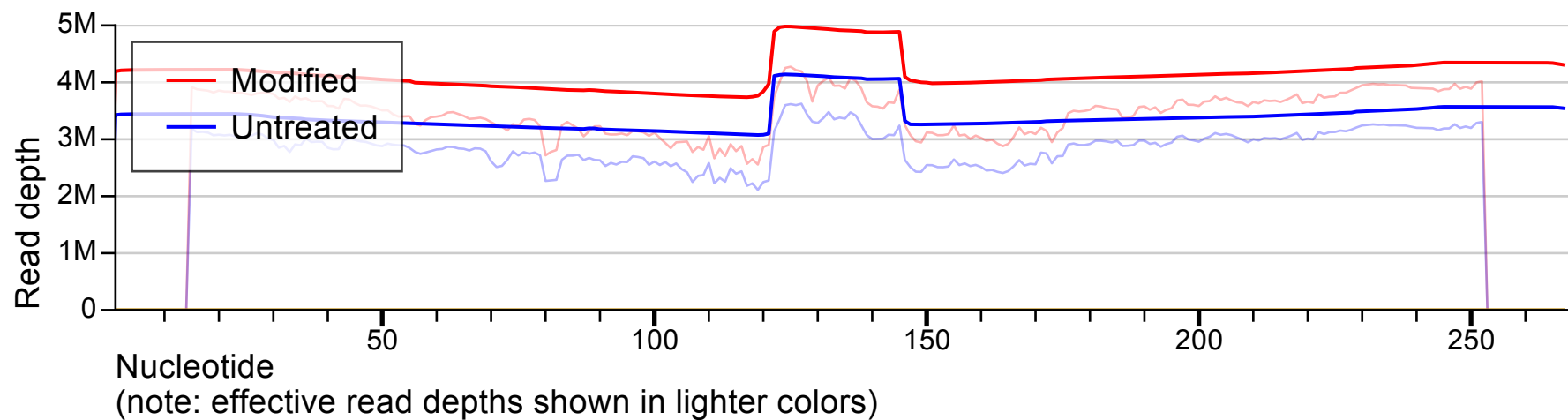

Supplement: Supplementary file 4 — Source Data [file 41467_2022_28295_MOESM4_ESM.zip › SourceData/RMRP-SHAPE-MaP/Wildtype_RMRP_1_RMRP_profiles.pdf]

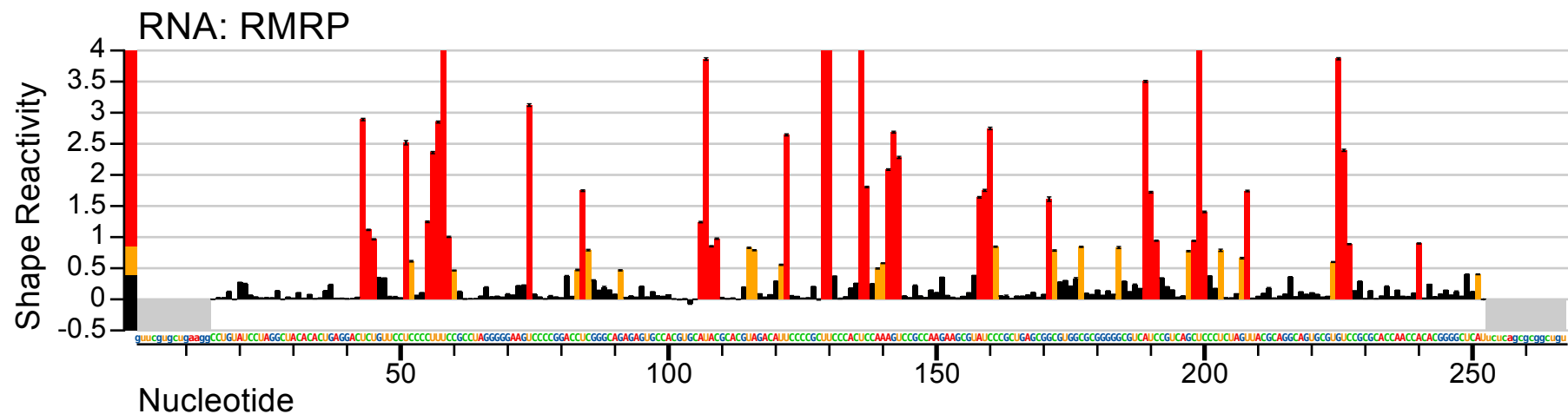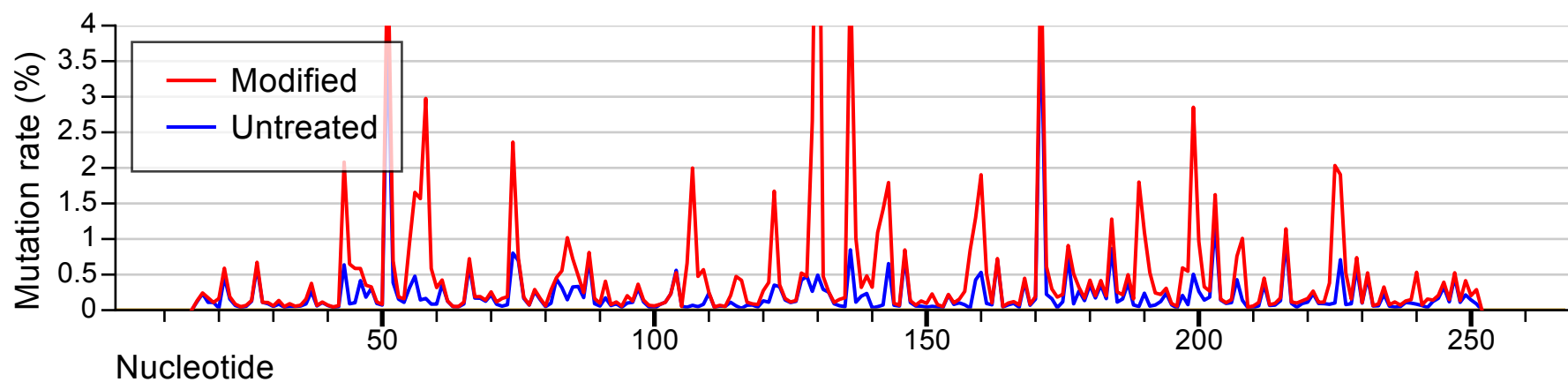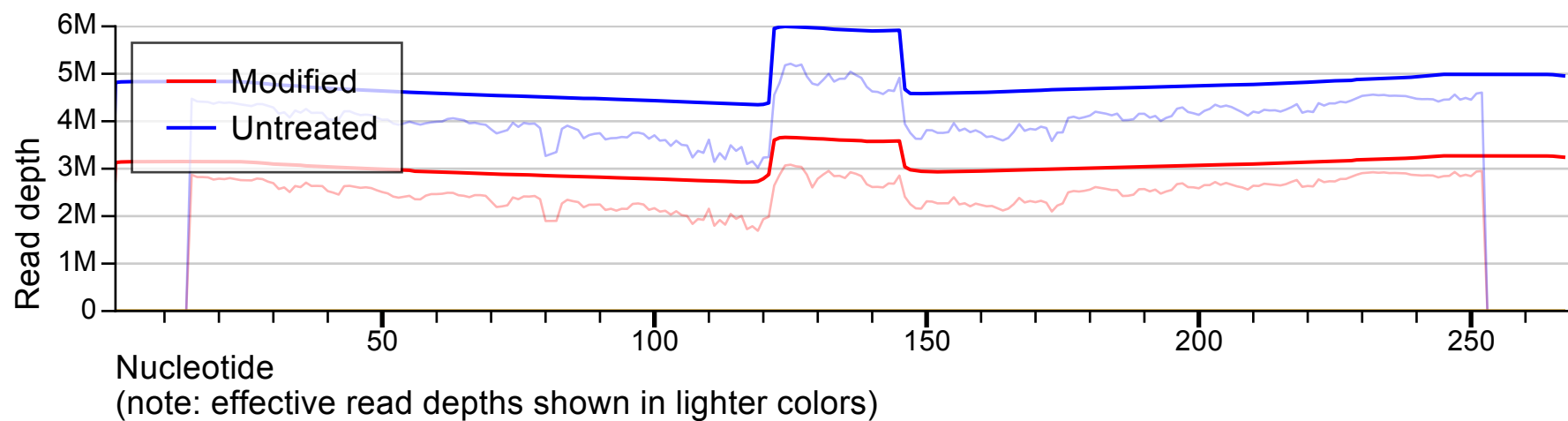

Supplement: Supplementary file 4 — Source Data [file 41467_2022_28295_MOESM4_ESM.zip › SourceData/RMRP-SHAPE-MaP/70AG_RMRP_1_RMRP_profiles.pdf]

# RNA: RMRP

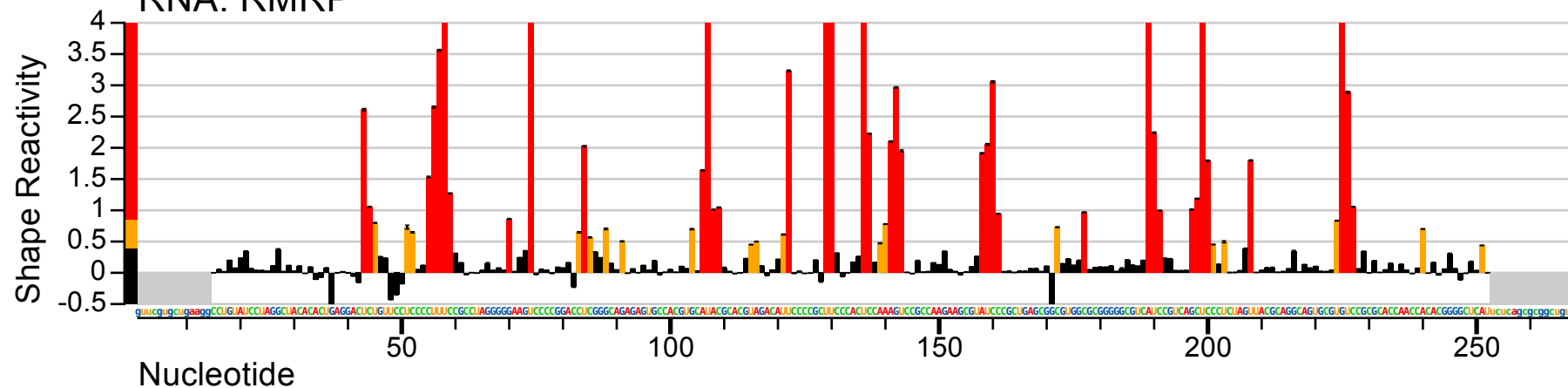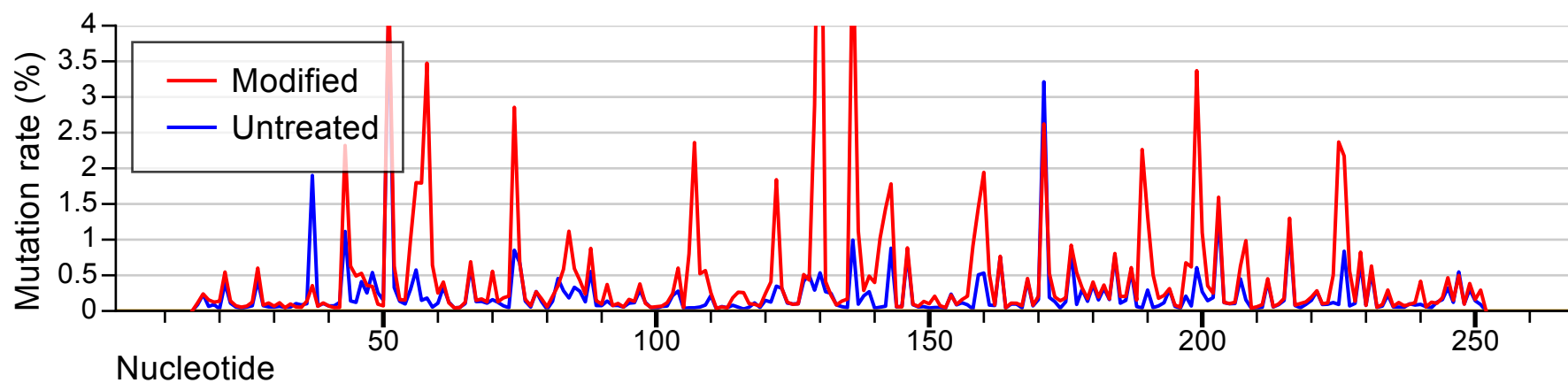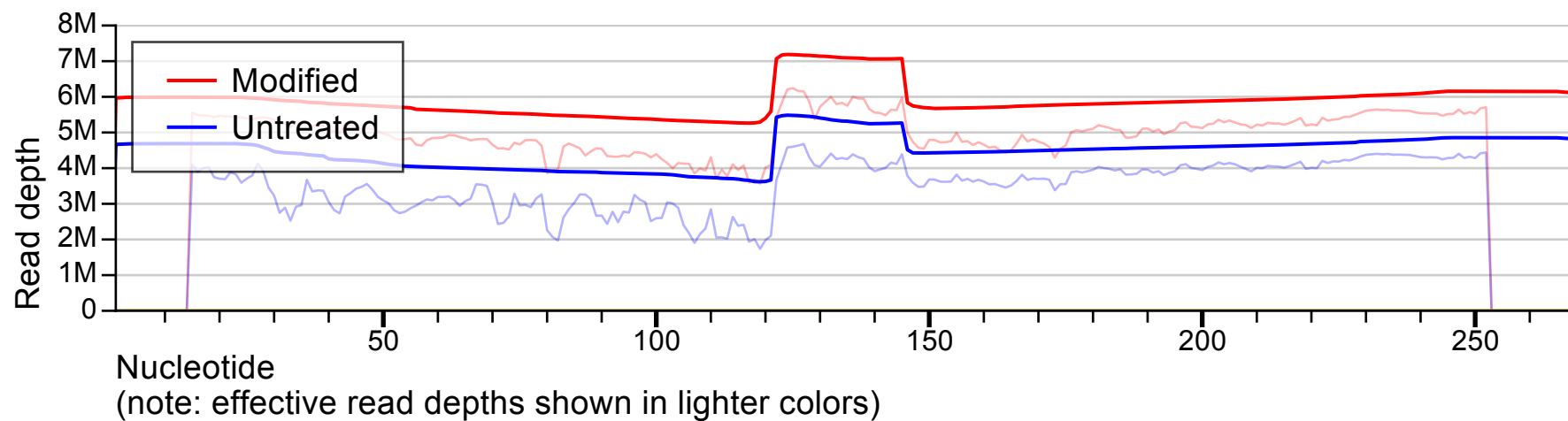

Supplement: Supplementary file 4 — Source Data [file 41467_2022_28295_MOESM4_ESM.zip › SourceData/RMRP-SHAPE-MaP/70AG_RMRP_4_RMRP_profiles.pdf]

$\Delta$ SHAPE

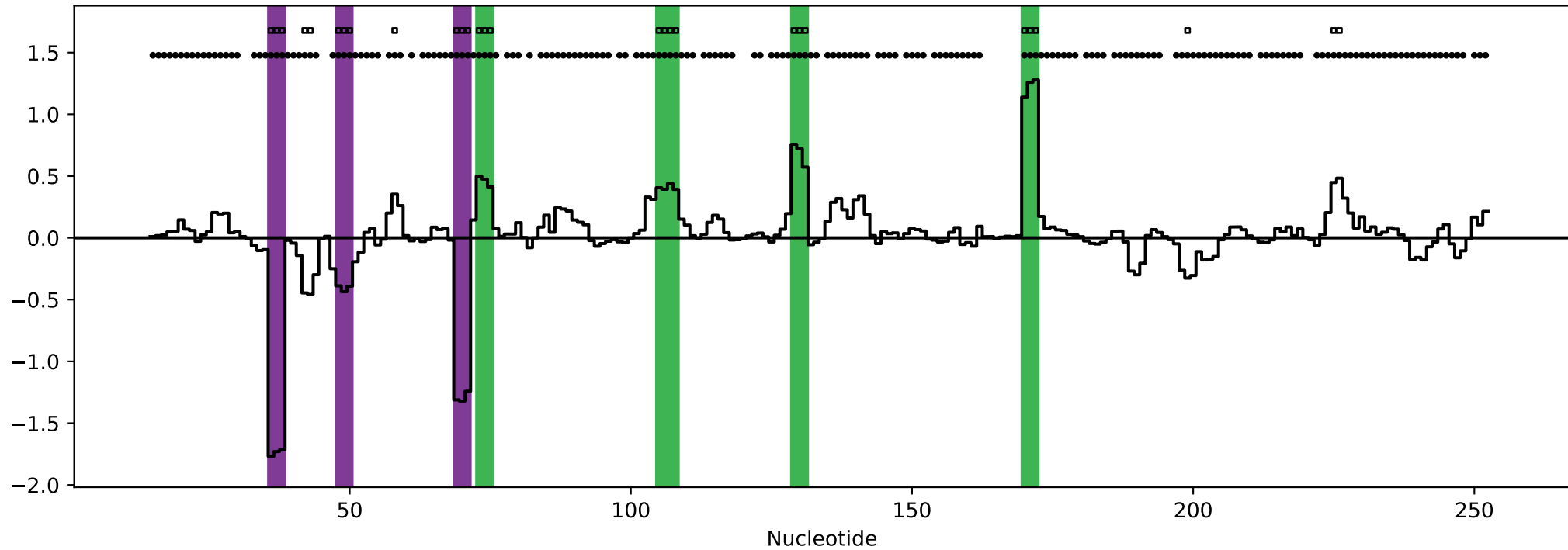

Supplement: Supplementary file 4 — Source Data [file 41467_2022_28295_MOESM4_ESM.zip › SourceData/RMRP-SHAPE-MaP/70AG3vsWT2.pdf]

$\Delta$ SHAPE

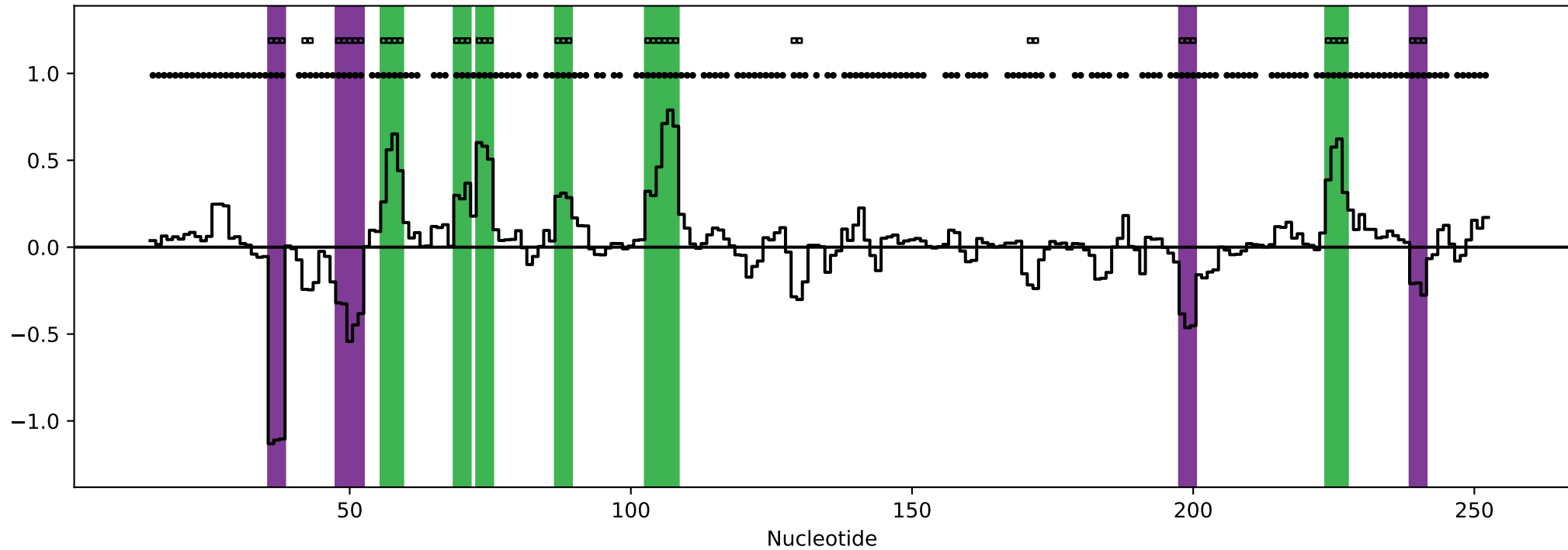

Supplement: Supplementary file 4 — Source Data [file 41467_2022_28295_MOESM4_ESM.zip › SourceData/RMRP-SHAPE-MaP/70AG4vsWT3.pdf]

Note: possible data quality issue - see log file

RNA: RMRP

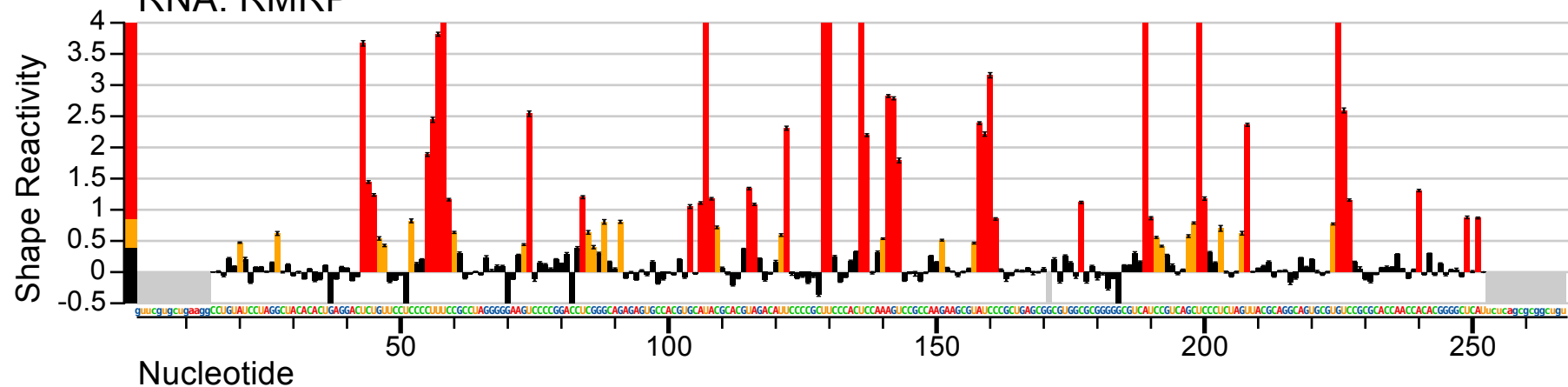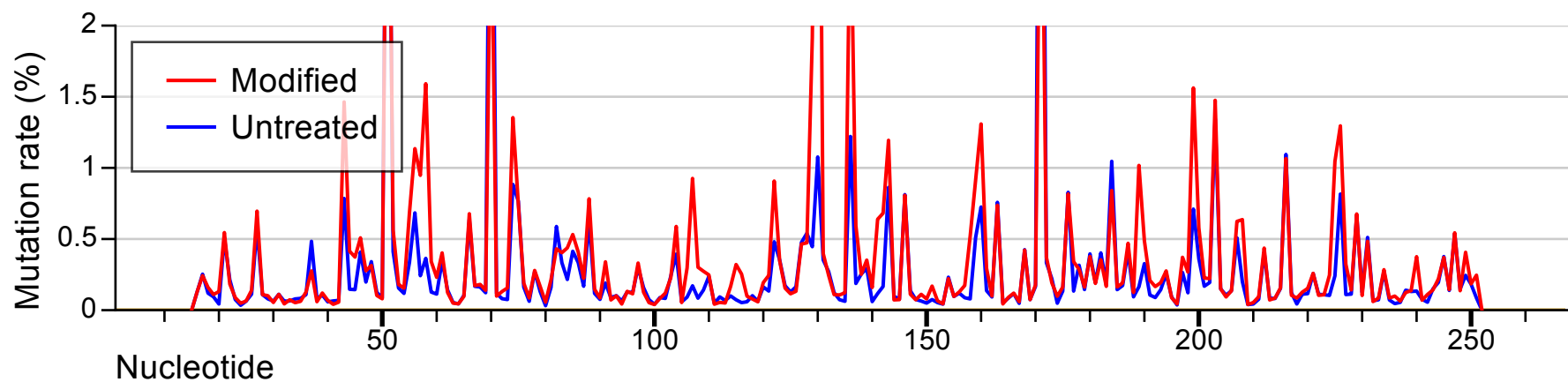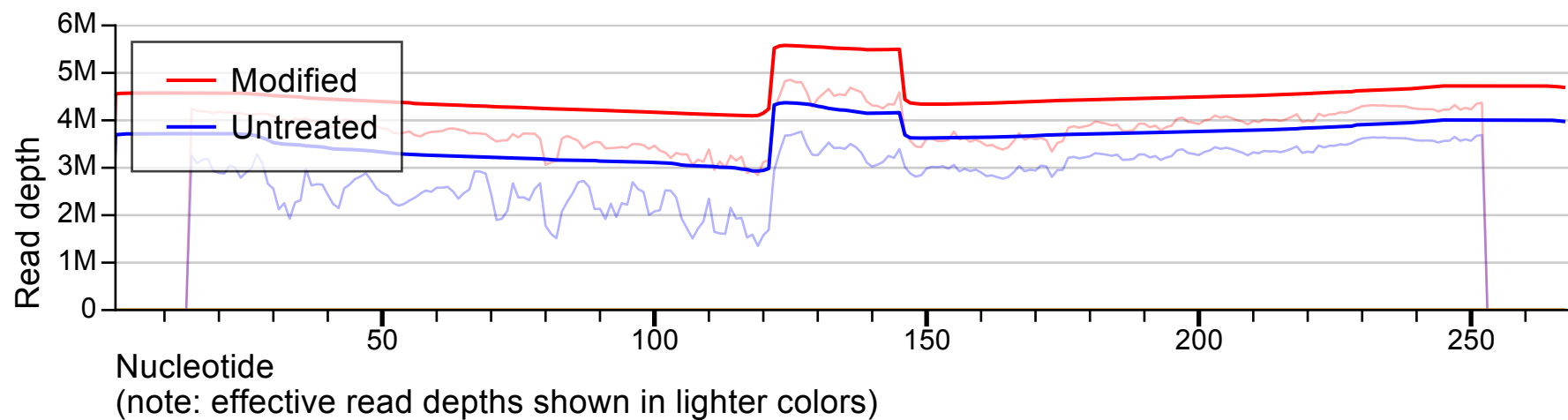

Supplement: Supplementary file 4 — Source Data [file 41467_2022_28295_MOESM4_ESM.zip › SourceData/RMRP-SHAPE-MaP/70AG_RMRP_2_RMRP_profiles.pdf]
