# Supplementary material for: A disease-linked lncRNA mutation in RNase MRP inhibits ribosome synthesis
Source: Nat Commun. 2022 Feb 3;13:649. doi: 10.1038/s41467-022-28295-8 (PMC8814244; doi:10.1038/s41467-022-28295-8)
Supplement: Supplementary file 4 — Source Data [file 70AG_RMRP_3_RMRP_profiles.pdf › SourceData/RMRP-SHAPE-MaP/70AG_RMRP_3_RMRP_profiles.pdf]

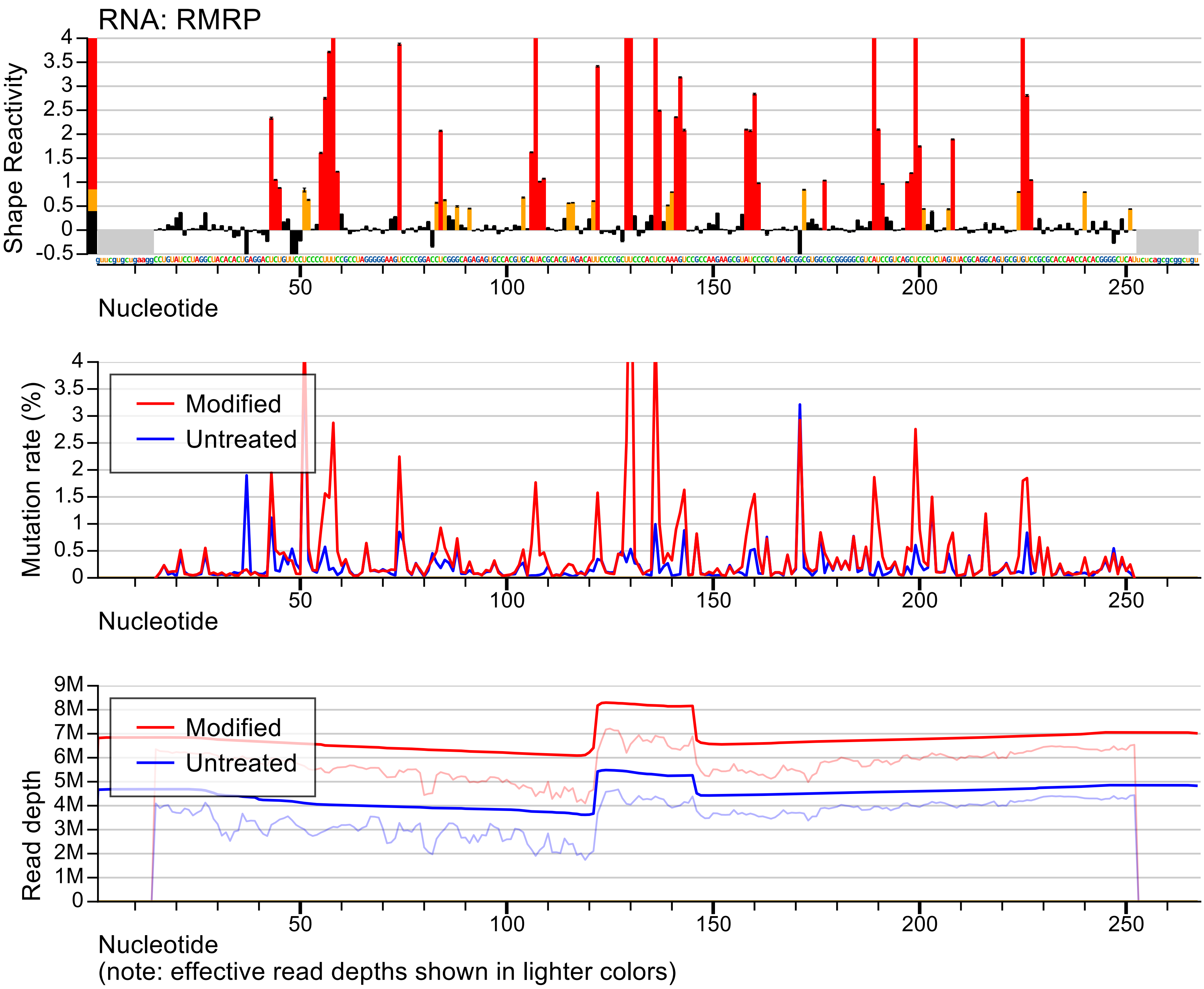
RNA: RMRP
Shape Reactivity
4
3.5
3
2.5
2
1.5
1
0.5
0
-0.5
50
100
150
200
250
Nucleotide
Mutation rate (%)
Modified
Untreated
Nucleotide
Read depth
9M
8M
7M
6M
5M
4M
3M
2M
1M
0
Modified
Untreated
Nucleotide
(note: effective read depths shown in lighter colors)
